# PACS deficiency disrupts Golgi architecture and causes cytokinesis failures and seizure-like phenotype in *Drosophila melanogaster*

**DOI:** 10.1098/rsob.240267

**Published:** 2025-02-26

**Authors:** Anna Frappaolo, Gianluca Zaccagnini, Maria Giovanna Riparbelli, Gianni Colotti, Giuliano Callaini, Maria Grazia Giansanti

**Affiliations:** ^1^ Istituto di Biologia e Patologia Molecolari del CNR, Dipartimento di Biologia e Biotecnologie, Università Sapienza di Roma, Piazzale A. Moro 5, 00185, Roma, Italy; ^2^ Dipartimento di Scienze della Vita, Università di Siena, Via Aldo Moro 2, 53100, Siena, Italy; ^3^ Istituto di Biologia e Patologia Molecolari del CNR, Dipartimento di Scienze Biochimiche ‘A. Rossi-Fanelli’, Università Sapienza di Roma, Piazzale A. Moro 5, 00185, Roma, Italy

**Keywords:** *Drosophila*, cytokinesis, membrane trafficking, model organism, PACS, neurodevelopmental disease

## Abstract

The PACS (phosphofurin acidic cluster sorting protein) proteins are membrane trafficking regulators, required for maintaining cellular homeostasis and preventing disease states. Mutations in human *PACS1* and *PACS2* cause human neurodevelopmental disorders, characterized by epileptic seizures and neurodevelopmental delay. In vertebrates, functional analysis of PACS proteins is complicated by the presence of two paralogues which can compensate for the loss of each other. Here, we characterize the unique fly homologue of human PACS proteins. We demonstrate that Drosophila PACS (dPACS) is required for cell division in dividing spermatocytes and neuroblasts. In primary spermatocytes, dPACS colocalizes with GOLPH3 at the Golgi stacks and is essential for maintaining Golgi architecture. In dividing cells, dPACS is necessary for central spindle stability and contractile ring constriction. dPACS and GOLPH3 proteins form a complex and are mutually dependent for localization to the cleavage site. We propose that dPACS, by associating with GOLPH3, mediates the flow of vesicle trafficking that supports furrow ingression during cytokinesis. Furthermore, loss of dPACS leads to defects in tubulin acetylation and severe bang sensitivity, a phenotype associated with seizures in flies. Together our findings suggest that a Drosophila *PACS* disease model may contribute to understanding the molecular mechanisms underpinning human PACS syndromes.

**Figure:**
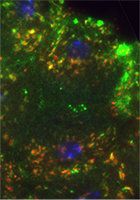

## 1. Introduction

The PACS (phosphofurin acidic cluster sorting protein) family was originally discovered in a genetic screen for cytosolic sorting proteins, that regulate trans-Golgi network (TGN) localization of the endoprotease furin and mannose-6-phosphate receptor (CI-MPR) by binding to specific phosphorylated acidic clusters [1,2]. The PACS family genes first appear in lower metazoan and are conserved from invertebrates to humans [3]. Invertebrates, including *Caenorhabitis elegans* and *Drosophila melanogaster,* harbour a single *PACS* gene whereas vertebrates express two paralogues namely PACS1 and PACS2 [4,5]. Functional analysis in different model systems revealed the requirement for PACS proteins for membrane trafficking, interorganellar communication and apoptosis [3,4]. Studies in human cell lines and transgenic mice showed that the Furin-binding region (FBR) of PACS1 and PACS2 enables interaction with several cargo molecules, mediating a variety of membrane trafficking itineraries [4]. The role of human PACS proteins in vesicle trafficking requires the formation of ternary complexes with other proteins which include the vesicular coat coatomer protein complex-I (COPI), the clathrin adaptors AP-1 and GGA3 (Golgi associated, gamma adaptin ear containing, ARF binding protein 3) [6–9]. PACS1, which interacts with AP-1 and GGA3, regulates endosome-to-TGN trafficking of furin [1,10] and other cellular proteins which include, among others, CI-MPR [8], polycystin-2 [7], VAMP-4 [11], sorting-related receptor with A-type repeats (SORLA) [12,13] and envelope glycoproteins from several pathogenic viruses [14,15]. Additionally, PACS1 is required for TGN localization of acidic cluster-containing client proteins to the primary cilium in epithelial cells [4,16,17]. Besides controlling membrane trafficking, PACS1 has been associated with the class IIb deacetylase HDAC6 which deacetylates alpha tubulin and maintains Golgi architecture [18].

PACS2 interacts with COPI and has been involved in Golgi to endoplasmic reticulum (ER) retrieval of several cargo molecules such as profurin, polycystin 2 and calnexin-2 [7,9,10,19].

PACS2 and *Caenorhabditis elegans* PACS (cePACS) also direct trafficking from early endosomes [20,21]. cePACS localizes to presynaptic endosomes where it mediates synaptic transmission [20]. PACS2 interacts with the metalloproteinase ADAM17 on early endosomes and controls ADAM17 recycling and cell surface availability which sustains the shedding of the epidermal growth factor receptor (EGFR) ligands [21]. Additionally, PACS2 plays a crucial function to integrate secretory trafficking with ER–mitochondria communication and TRAIL-induced apoptosis [22,23].

The human *PACS1* (*hPACS1*) locus has been linked to early-onset obesity [24] and developmental delay [3]. In addition, both hPACS1 and hPACS2 have been associated with human neurological disorders [25–28]. Mutations in *hPACS1* cause the Schuurs–Hoeijmakers syndrome (SHMS), also known as *PACS1* neurodevelopmental disorder (PACS1-NDD), a rare autosomal dominant disease characterized by epileptic seizures, autism, cerebellar abnormalities, distinctive craniofacial features, cryptorchidism in males and congenital abnormalities including heart and ocular defects and hypotonia [25–27]. Of note, consistent with the craniofacial anomalies of PACS1-NDD patients, injection of the mutant *hPACS1* mRNA in zebrafish embryos results in defects of cranial cartilaginous structures [25]. Individuals carrying a missense PACS2 variant, clinically present with neonatal/early infantile developmental and epileptic encephalopathies with or without autism, common cerebellar dysgenesis and facial dysmorphism [28].

In this study, we have characterized the single Drosophila orthologues of human PACS proteins. Mutants carrying null alleles of Drosophila *PACS* (*dPACS*) are viable but male sterile. We provide the first evidence for the role of PACS in cytokinesis. Loss of dPACS disrupts Golgi architecture and impairs central spindle structure and contractile ring constriction in dividing cells. Consistent with the phenotypic defects, dPACS localizes to the Golgi stacks in interphase spermatocytes and accumulates at the cleavage site during telophase. Furthermore, we dissect the relationship of dPACS with other proteins required for membrane trafficking in Drosophila cytokinesis. Based on our findings, dPACS cooperates with *Drosophila* GOLPH3 (dGOLPH3 [29]) and conserved oligomeric Golgi (COG) complex [30] to regulate the flow of vesicle trafficking that supports furrow ingression during cytokinesis. Finally, we show that *dPACS* mutant flies display defects in tubulin acetylation and severe bang sensitivity, a phenotype associated with seizures in flies.

## 2. Results

### 2.1. The Drosophila homologue of human phosphofurin acidic cluster sorting protein is required for cytokinesis

**Figure 1.**
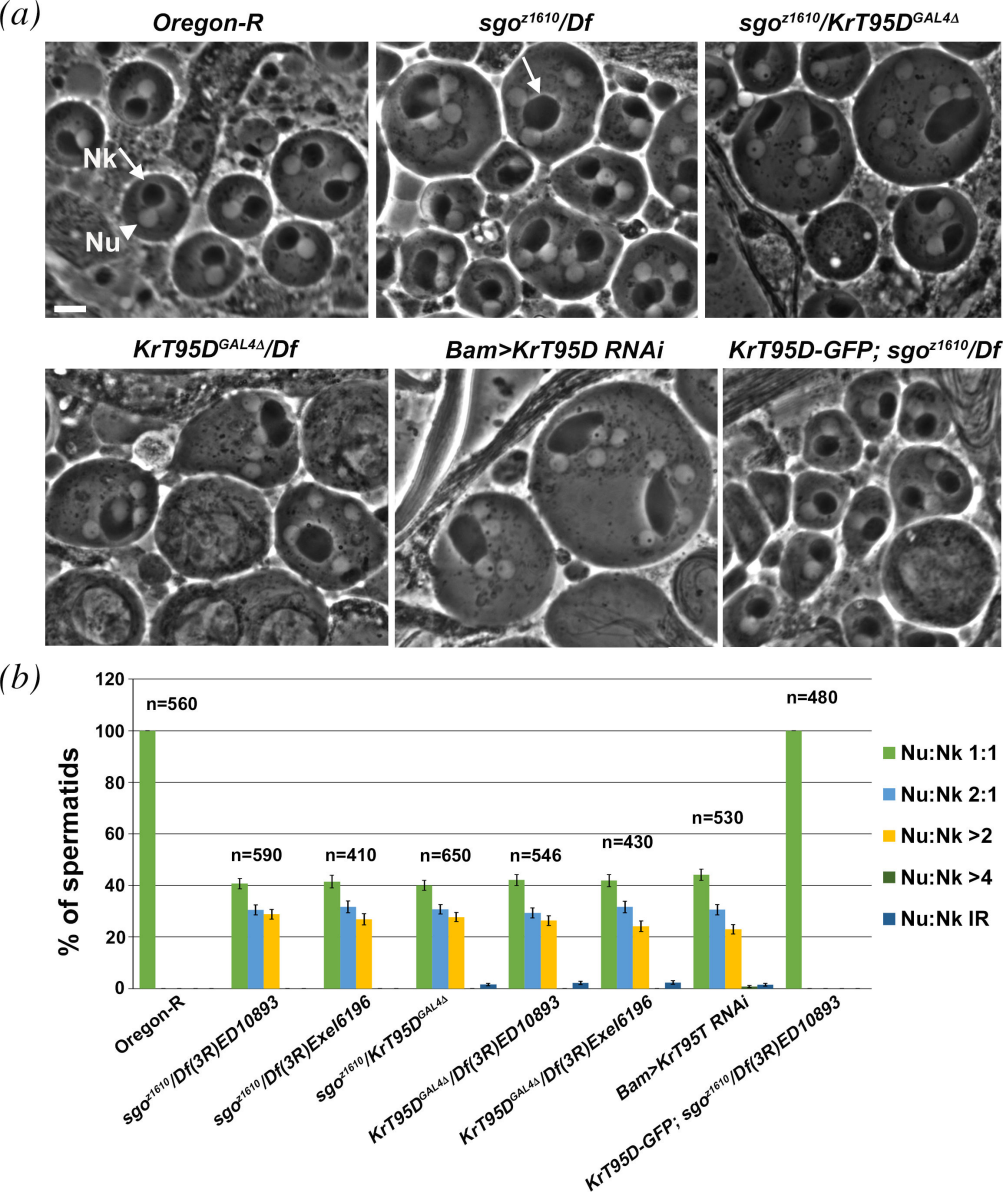
dPACS is required for male meiotic cytokinesis. (*a*) Phase-contrast micrographs of onion stage spermatids. Each wild-type spermatid exhibits a single nucleus (Nu, white arrowhead) and a single phase-dark mitochondrial derivative (Nebenkern, Nk, white arrow), of similar size. Cytokinesis failures during male meiosis of *KrT95D* mutants (*sgo^z1610^*/*Df(3R)ED10893*; *sgo^z1610^*/*KrT95D^GAL4Δ^* and *KrT95D^GAL4Δ^*/*Df(3R)ED10893,* respectively) and in testes from males expressing *UAS::KrT95DRNAi* under the control of *Bam-GAL4* (*Bam>KrT95DRNAi*) result in aberrant spermatids containing multiple nuclei associated with enlarged nebenkern (white arrow). A single copy of *GFP-KrT95D* transgene rescues cytokinesis defects of *sgo^z1610^*/*Df(3R)ED10893 (sgo^z1610^*/*Df*) mutants. Scale bar: 10 μm. (*b*) Frequencies of spermatids containing 1 or multiple nuclei per mitochondrial derivative in wild-type (Oregon-R), in testes from either *KrT95D* mutant males or *Bam>KrT95DRNAi* and in testes of genotype *GFP-KrT95D; sgo^z1610^*/*Df(3R)ED10893*. Data are percentage ± s.e.; *n* = total number of spermatids examined from testes of at least 10 males.

The Drosophila *smeagol* allele (*sgo^z1610^*) was identified during a screen for mutations causing male sterility and defective spermatocyte cytokinesis and located to the chromosomal interval *95A5-95D11* defined by the deletion *Df(3R)mbc-R1* [31]. Complementation analysis with a series of chromosomal deficiencies uncovering *95A5-95D11*, revealed that *sgo^z1610^* failed to complement both *Df(3R)ED10893* and *Df(3R)Exel6196* for spermatocyte cytokinesis defects, indicating that it maps to a genomic region that contained the *Krueppel target at 95D* (*KrT95D*) gene (figure 1*a*,*b*). *KrT95D^GAL4Δ^*, a null allele of *KrT95D,* in which the entire coding sequence of the gene is replaced by GAL4, failed to complement *sgo^z1610^* for male sterility and cytokinesis failures (figure 1*a*,*b*). Consistent with these results, *KrT95D^GAL4Δ^* was uncovered by both *Df(3R)ED10893* and *Df(3R)Exel6196* (figure 1*a*,*b*). Finally, testes from males expressing double-stranded RNA (dsRNA) against *KrT95D* in spermatocytes, displayed frequent multinucleate spermatids indicating cytokinesis failures comparable to *sgo^z1610^* and *KrT95D^GAL4Δ^* mutants (figure 1*a*,*b*). GFP-KrT95D protein expressed under the control of the α1-tubulin promoter, fully rescued male sterility and the cytokinesis defects of *sgo^z1610^*/*Df(3R)ED10893* mutant males, confirming that the cytokinesis phenotype is the consequence of mutation in the *KrT95D* gene (figure 1*a*,*b*). DNA sequencing revealed that the EMS-induced *sgo^z1610^* mutant allele carried a nonsense mutation in the *KrT95D* gene encoding a polypeptide of predicted 124.5 kDa, which belongs to a family of endomembrane trafficking molecules known as PACS proteins (electronic supplementary material, figures S1 and S2). Thus, hereafter, we refer to *KrT95D* as Drosophila *PACS (dPACS*). Search for homologies via the DRSC integrative orthologues prediction (DIOPT) tool (https://www.flyrnai.org/diopt [32]) indicated dPACS as the fly orthologues of human PACS1 and PACS2 (hPACS1 and hPACS2). This analysis revealed that the highest homology was between dPACS and hPACS2 protein (electronic supplementary material, figures S1 and S2). The alignments between dPACS and human PACS proteins are shown in the electronic supplementary material, figures S1 and S2 [33]. dPACS, hPACS1 and hPACS2 belong to a family of multifunctional trafficking proteins, which bind cargo containing acidic cluster sorting motifs and regulate secretory and endocytic pathways [4].

Data reported in FlyAtlas indicated expression of the *dPACS* gene in several tissues including adult testes and brains [34]. We used the *dPACS^GAL4Δ^* allele in which the entire coding sequence of *dPACS* is replaced by GAL4 (see above) and the UAS/GFP binary system to determine the expression pattern of *dPACS*. Crossing *dPACS^GAL4Δ^* animals with animals carrying the *UAS-GFP* transgene (used as reporter), revealed that the *dPACS* gene is expressed in multiple tissues of pupae and adults (electronic supplementary material, figure S3a,b). Immunostaining of pupal brains from *UAS-GFP; dPACS^GAL4Δ^*/*+* animals, for GFP and the nuclear neuronal marker Elav (Embryonic Lethal Abnormal Vision), revealed that *dPACS* is highly expressed in the pupal ventral nerve cord (electronic supplementary material, figure S3b).

**Figure 2.**
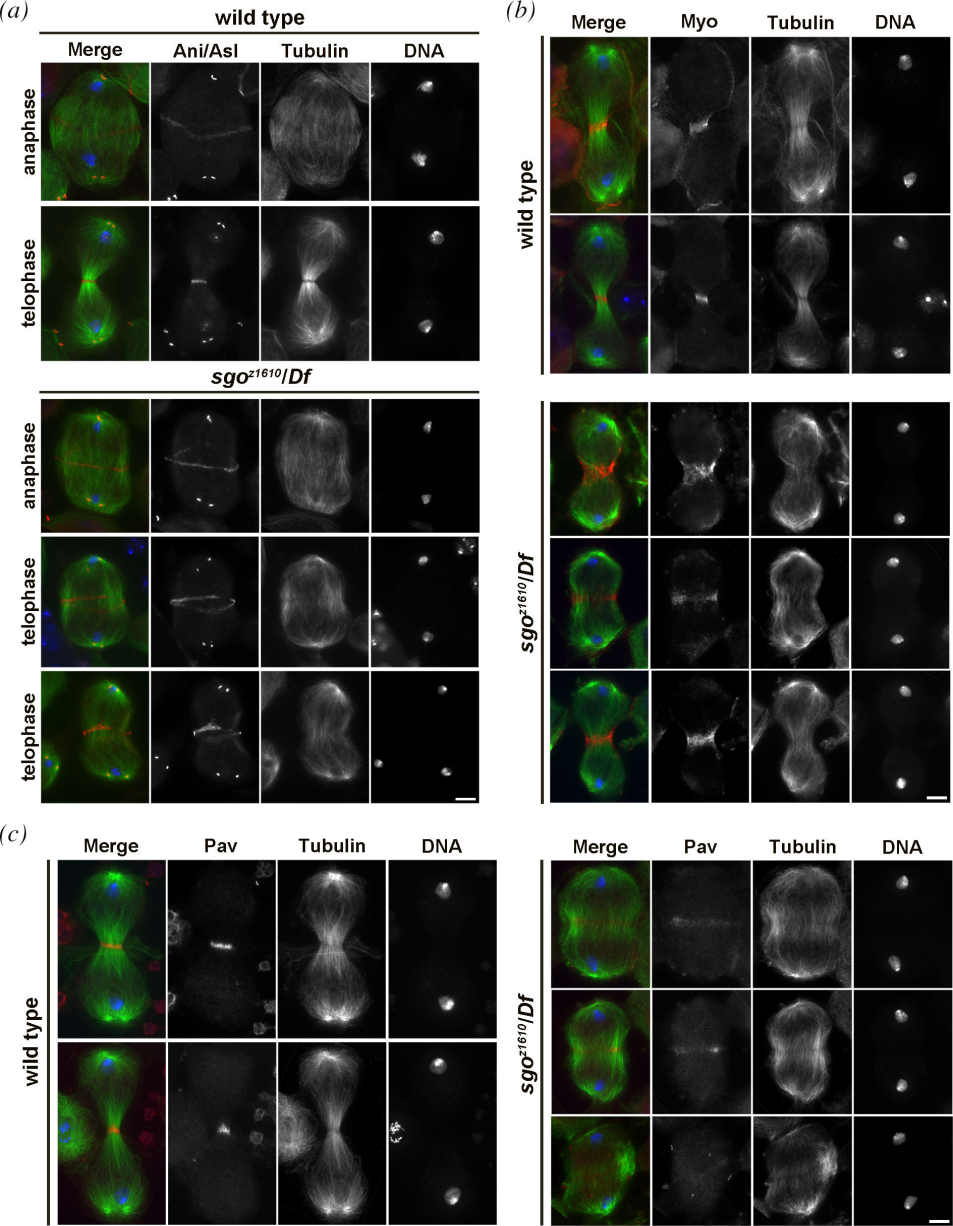
Dividing spermatocytes from *dPACS* mutants assemble defective contractile rings. (*a*) Dividing primary spermatocytes during late anaphase and mid- to late telophase were stained for tubulin (green), Anillin/Asterless (Ani/Asl, red) and DNA (blue). *n* = 40 wild-type and *sgo^z1610^/Df* late-anaphase/early telophase spermatocytes; *n* = 48 wild-type and *sgo^z1610^/Df* mid- to late telophase spermatocytes; randomly selected from images taken in five experiments. (*b*) Mid- to late telophase I spermatocytes stained for myosin II (Myo, red), tubulin (green) and DNA (blue). *n* = 50 wild-type and *sgo^z1610^/Df* mid- to late telophase spermatocytes; randomly selected from images taken in five experiments. (*c*) Mid- to late telophase I spermatocytes stained for Pavarotti (Pav, red), tubulin (green) and DNA (blue). *n* = 50 wild-type and *sgo^z1610^/Df* mid- to late telophase spermatocytes; randomly selected from images taken in five experiments. Scale bar: 10 μm.

Previous characterization of dividing spermatocytes revealed that *sgo^z1610^* displayed defective central spindle and assembled anillin rings that failed to constrict in late telophase [31]. Consistent with these data, all the dividing spermatocytes from wild-type and from *sgo^z1610^*/*Df(3R)ED10893 (sgo/Df*) mutant males formed anillin rings at the equatorial cortex during late anaphase and early telophase (figure 2*a*). However, during later stages of cytokinesis, 100% of wild-type mid- to late-telophase cells had constricted rings whereas 71% of *sgo/Df* mutant cells displayed large and unconstricted anillin rings (figure 2*a*).

To further substantiate the cytokinesis phenotype of *sgo/Df* mutants, dividing spermatocytes were stained for the non-muscle Myosin II (Myo II) heavy chain Zipper [35] (figure 2*b*). Loss of function of dPACS also resulted in failure to constrict the Myo II cortical rings, visualized by anti-Zipper antibodies, in 72% of mid- to late telophase spermatocytes (figure 2*b*). Localization of Pavarotti (Pav), the Drosophila orthologues of human MKLP1 (mitotic kinesin-like protein 1 [36]), was also perturbed in *sgo/Df* mutant spermatocytes. Mid-telophases from wild-type displayed a tight equatorial band of Pav (100% of dividing mid-telophases). Conversely, 76% of mid-telophases from *sgo/Df* mutants had only a faint concentration of Pav at both peripheral and interior microtubules (figure 2*c*). Overall, our findings revealed that the contractile ring components are recruited to the cleavage furrow of spermatocytes lacking dPACS but they fail to mediate completion of cytokinesis.

**Figure 3.**
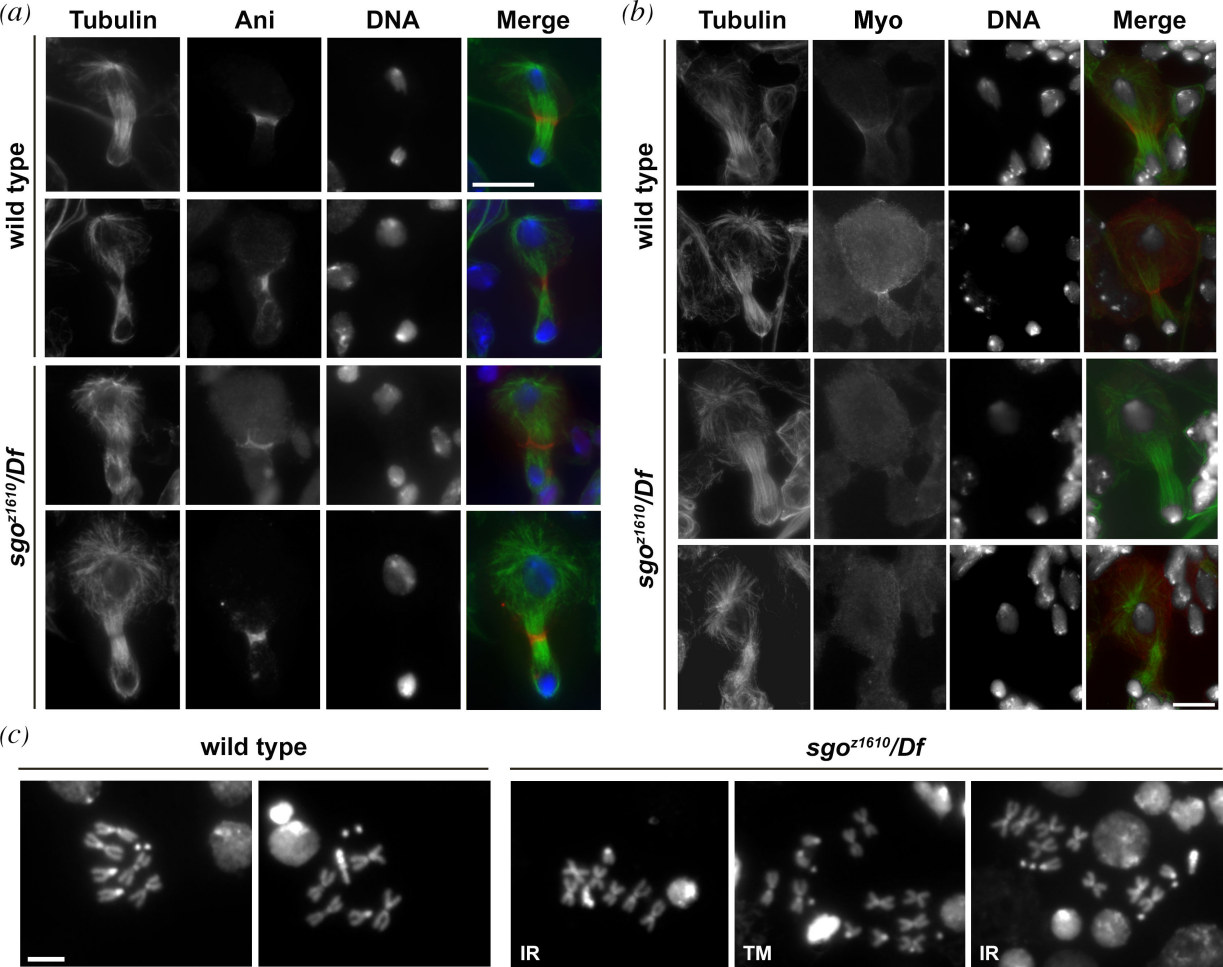
Loss of *dPACS* causes cytokinesis failures in larval neuroblasts. (*a*) Wild-type and *sgo^z1610^*/*Df(3R)ED10893 (sgo^z1610^*/*Df*) mutant NBs during mid- to late telophase, stained for tubulin (green), Anillin (Ani, red) and DNA (blue). *n* = 98 wild-type and *n* = 106 *sgo^z1610^/Df* mid- to late telophases randomly selected from images taken in five experiments. (*b*) Wild-type and *sgo^z1610^*/*Df(3R)ED10893 (sgo^z1610^*/*Df*) mutant NBs during mid- to late telophase, were stained for tubulin (green), myosin II (Myo, red) and DNA (grey). *n* = 88 wild-type and *n* = 92 *sgo^z1610^/Df* mid- to late telophase randomly selected from images taken in five experiments. (*c*) Normal metaphases from wild-type and irregular metaphases from *sgo^z1610^*/*Df* in larval central nervous system. IR, hyperploid metaphase; TM, tetraploid metaphase. The cells were randomly selected from images taken in five independent experiments. Scale bars: (*a,b*) 10 μm and (*c*) 5 μm.

Immunostaining of larval brains for tubulin and either anillin or MyoII revealed that wild-type function of dPACS is required for cytokinesis in dividing neuroblasts (NBs) (figure 3*a*,*b*). One hundred per cent of mid- to late telophase NBs from wild-type showed constricted anillin rings (figure 3*a*), whereas 34% of mid- to late telophase NBs from *sgo/Df* mutants had unconstricted anillin rings. Likewise, in all wild-type mid- to late telophase NBs, MyoII concentrated in constricted rings (100% of mid-telophases, figure 3*b*). In contrast, in 37% of mid-telophase NBs from *sgo/Df* mutants, MyoII failed to form constricted rings (figure 3*b*).

Analysis of larval brain squashes revealed a low frequency of tetraploid and hyperploid metaphases (3%, *n* = 270) compared to 0% in wild-type (*n* = 300; figure 3*c*).

### 2.2. dPACS protein localizes to Golgi organelles and to the cleavage furrow and is required for Golgi structure maintenance

**Figure 4.**
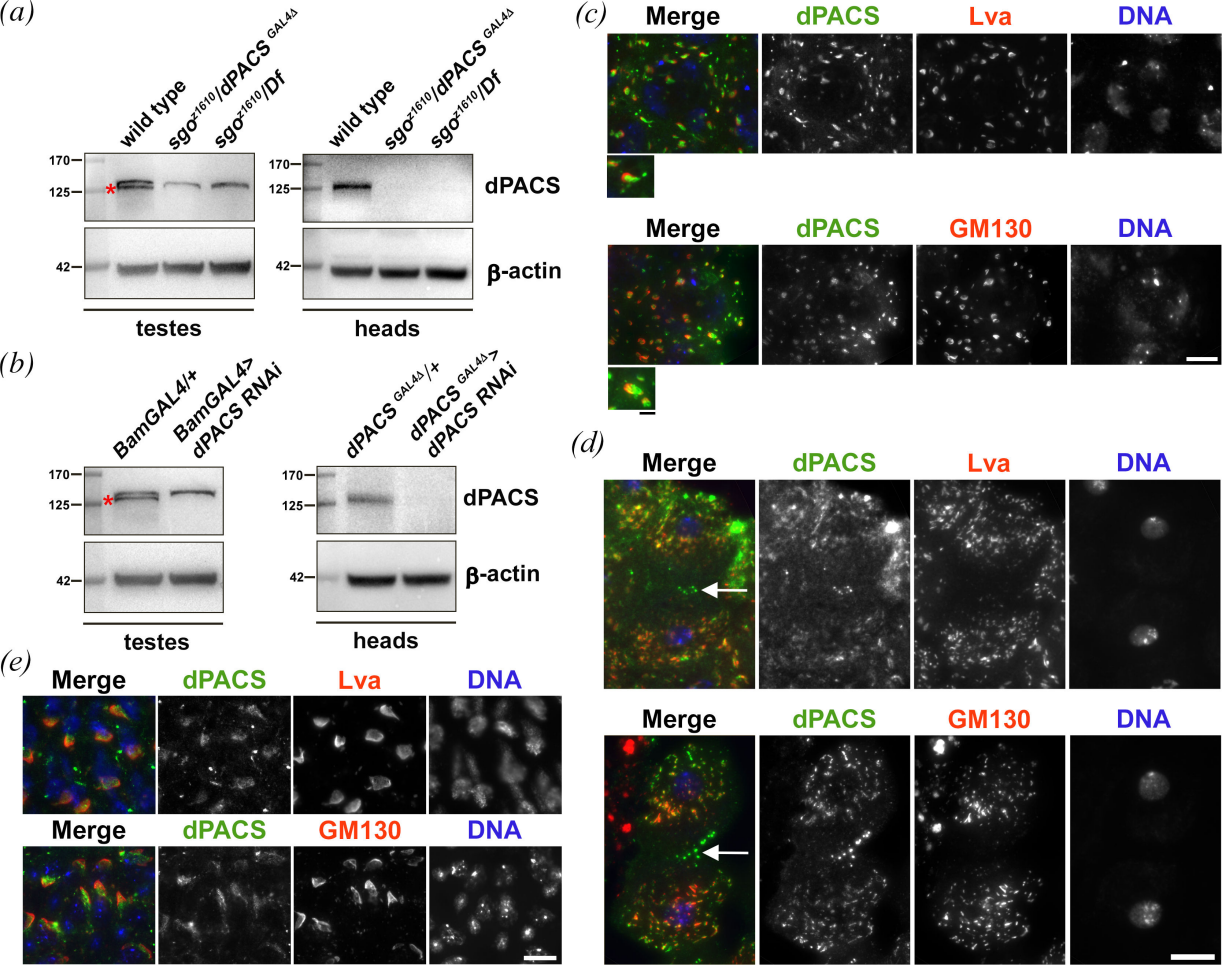
dPACS localizes to Golgi organelles in spermatocytes and spermatids. (*a,b*) WB from adult testis (testes) and head extracts (heads). Polyclonal rabbit anti-dPACS recognized a band of approximately 125 kDa. This band is absent in extracts from either *sgo^z1610^/dPACS^GAL4Δ^* or *sgo^z1610^/Df(3R)ED10893 (sgo^z1610^*/*Df*) mutants (*a*) and upon dPACS depletion in either testes or heads (*b*). β-actin was used as a loading control. Molecular weights are in kilodaltons. Note that in WB from testes the band that is absent from mutant extracts corresponds to dPACS protein (red asterisks); the upper band is not specific. WB experiments in (*a,b)* were performed three times with identical results. (*c*) Colocalization of dPACS (green) with the Golgi proteins Lva and GM130 (red) and DNA (blue) in interphase spermatocytes. Enlarged panels show colocalization or partial colocalization of proteins at the Golgi. (*d*) Telophase spermatocytes were stained for dPACS (green), DNA (blue) and either Lva or GM130 (red). White arrows point to dPACS accumulation at the cleavage site. (*e*) Onion stage spermatids stained for dPACS (green), DNA (blue) and either Lva, or GM130 (red). (*c–e*) Scale bars: 10 and 2.5 μm in the inset; *n* = 30 cells were analysed for each double staining. The cells were randomly selected from images taken in five independent experiments.

Polyclonal antibodies raised against dPACS protein recognized a band of the expected size (120 kDa) in western blots (WB) from testis and fly head extracts (figure 4*a*). This band was not detected in extracts from both *sgo ^z1610^*/*Df* and *sgo ^z1610^*/*dPACS^GAL4Δ^* mutant flies (figure 4*a*) and in testes and heads expressing dsRNA against *dPACS*, indicating that the antibodies specifically reacted with dPACS protein (figure 4*a*,*b*). As expected, anti-dPACS antibodies reacted with GFP-dPACS protein in WB from flies expressing GFP-dPACS (electronic supplementary material, figure S4a).

Immunofluorescence analysis revealed that both the endogenous dPACS and GFP-tagged dPACS proteins localized to Golgi organelles in primary spermatocytes (figure 4*c*; electronic supplementary material, figure S4b). In primary spermatocytes dPACS was enriched at multiple round structures (figure 4*c*) that also contained the Golgi proteins Lava lamp (Lva [37]; figure 4*c*), Golgi matrix protein 130 kD (GM130; figure 4*c*), the COG complex subunit Cog7 [30,38] and the phosphatidylinositol 4-phosphate [PI(4)P] effector dGOLPH3 [29] (electronic supplementary material, figure S5a,b). hPACS1 was shown to bind AP-1 complexes [1,6]. Previous studies indicated that AP-1 localized to the Golgi stacks of Drosophila Dmel2 cells, where it was mainly enriched in the trans-Golgi/TGN [39]. Co-staining of male meiotic cells for AP-1 and dPACS revealed that AP-1 and dPACS proteins did not show precise colocalization at the Golgi stacks of interphase spermatocytes, indicating that AP-1 may reside mostly at the trans-Golgi/TGN, whereas dPACS may localize throughout the Golgi (electronic supplementary material, figure S6a). Aside from localizing to Golgi organelles, dPACS protein was also enriched in Rab4-positive endosomes in interphase spermatocytes expressing GFP-Rab4 (electronic supplementary material, figure S5c).

**Figure 5.**
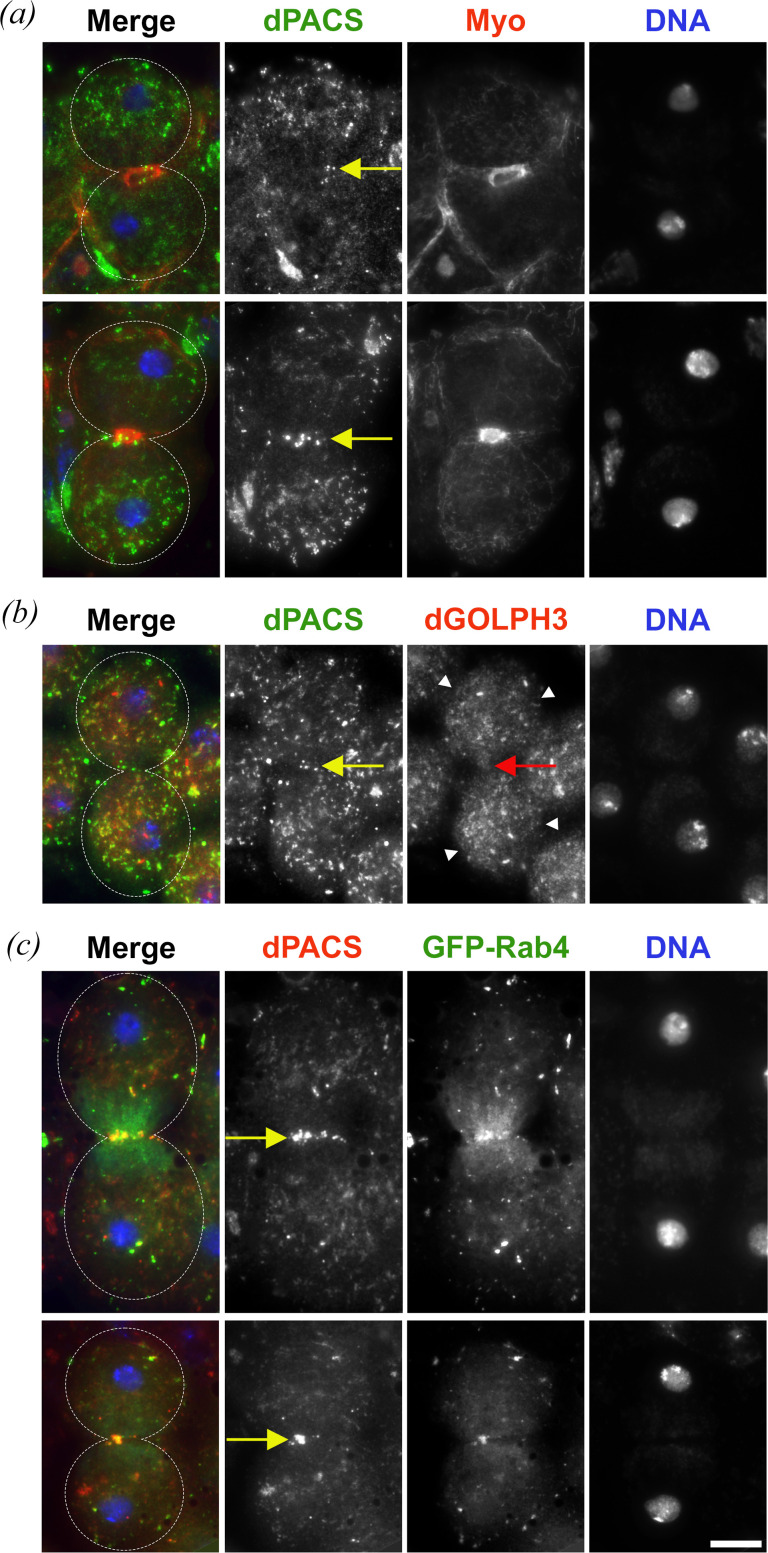
dPACS localization in telophase spermatocytes. (*a,b*) Telophase spermatocytes were stained for dPACS (green), DNA (blue) and either MyoII (red) or dGOLPH3 (red). (*c*) Telophase spermatocytes expressing GFP-Rab4 were stained for GFP (green), dPACS (red) and DNA (blue). (*a–c*) *n* = 30 cells were analysed for each double staining. Yellow arrows point to dPACS accumulation at the cleavage site; red arrow points to dGOLPH3 accumulation at the equatorial site, arrowheads indicate dGOLPH3 signals at the cell poles. The dot lines indicate the cell boundaries. The cells were randomly selected from images taken in five independent experiments. Scale bar: 10 μm.

Similar to other Golgi proteins (Lva, AP-1, GM130) most dPACS protein was concentrated around the polar regions of dividing spermatocytes (figure 4*d*; electronic supplementary material, figure S6a). Additionally, dPACS signals (but not Lva, GM130 and AP-1), were also enriched at the midzone of telophase spermatocytes (figure 4*d*; electronic supplementary material, figure S6a). To better analyse localization of dPACS at the cleavage site, telophase spermatocytes were stained with anti-dPACS and either anti-MyoII or anti-GOLPH3 antibodies (figure 5*a*,*b*). Additionally, dividing spermatocytes expressing GFP-Rab4 were immunostained for GFP and dPACS (figure 5*c*). Overall, these results showed that dPACS-enriched puncta overlap with GFP-Rab4 at the cell equator during cytokinesis, suggesting that dPACS protein localizes to vesicles accumulating at the cleavage furrow (figure 5). Due to technical limitations of the immunostaining protocol for pupal brains, we could not visualize dPACS-enriched vesicles in dividing NBs. However, immunofluorescence analysis of telophase NBs revealed that dPACS protein is dispersed in the cytoplasm and localizes to the spindle midzone with a punctate pattern (electronic supplementary material, figure S3c).

In wild-type early round spermatids, we visualized dPACS protein with Lva, GM130 and AP-1 in the acroblast, a specialized Golgi assemblage, comparable to the mammalian Golgi ribbon and located in close proximity to the nucleus (figure 4*e*; electronic supplementary material, figure S6a).

**Figure 6.**
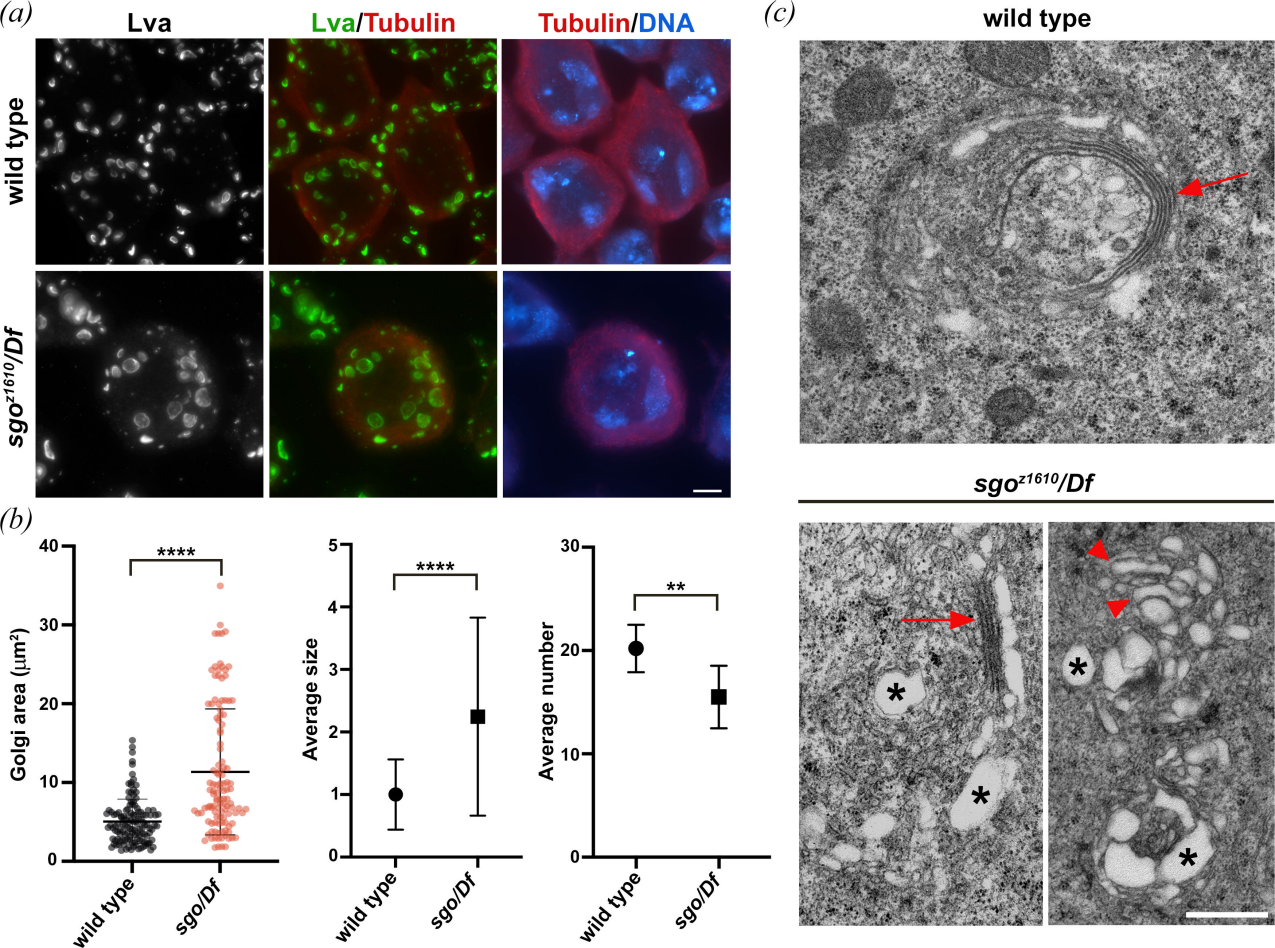
Loss of dPACS disrupts Golgi architecture in interphase spermatocytes. (*a*) Interphase spermatocytes stained for DNA (blue), Lva (green) and tubulin (red). Scale bar: 10 μm. (*b*) Lva-positive bodies were analysed using the ImageJ software in wild-type and *sgo^z1610^/Df* mutant spermatocytes. In total, *n* = 128 Golgi from wild-type and *n* = 117 Golgi from *sgo^z1610^/Df* mutant spermatocytes were examined to measure the Golgi area (scatter plot indicating mean ± s.d. is shown on the left). Graphs represent respectively the average size (relative to wild-type ± s.d.) and average number ( ±s.d.) of Lva-positive bodies. *n* = 35 wild-type and *sgo^z1610^/Df* cells were examined, randomly selected from images taken in five independent experiments. Statistically significant differences are ****p < 0.0001; **p < 0.01 (Mann–Whitney *U*-test). (*c*) Transmission electron micrographs showing Golgi complexes in primary spermatocytes from wild-type and *sgo^z1610^/Df* mutants. Arrow in the wild-type indicates a normal Golgi stack. Golgi compartments in *sgo^z1610^/Df* mutants are swollen and vacuolated (asterisks), with enlarged cisternae (arrowhead) and reduced stacking (arrow). Scale bar: 500 nm.

Our previous work identified several membrane-trafficking proteins required for the proper structure of Golgi stacks in fly male meiotic cells [29,30,40–42]. Likewise, dPACS is essential for maintaining Golgi architecture in primary spermatocytes. In interphase wild-type spermatocytes stained for the golgin Lva, the average number of fluorescent bodies per cell was 20 (figure 6*a*,*b*). Loss of dPACS reduced the number and altered the size of Lva-spherical structures in interphase spermatocytes (figure 6*a*,*b*). In primary spermatocytes from *sgo/Df* mutant males, analysed by transmission electron microscopy (TEM), the ultrastructure of the Golgi apparatus was severely disrupted. The Golgi compartments of mutant cells appeared swollen and vacuolated, with enlarged cisternae and reduced stacking (figure 6*c*).

### 2.3. Subcellular localization of dPACS at the Golgi requires wild-type function of Cog7 and dGOLPH3 proteins

**Figure 7.**
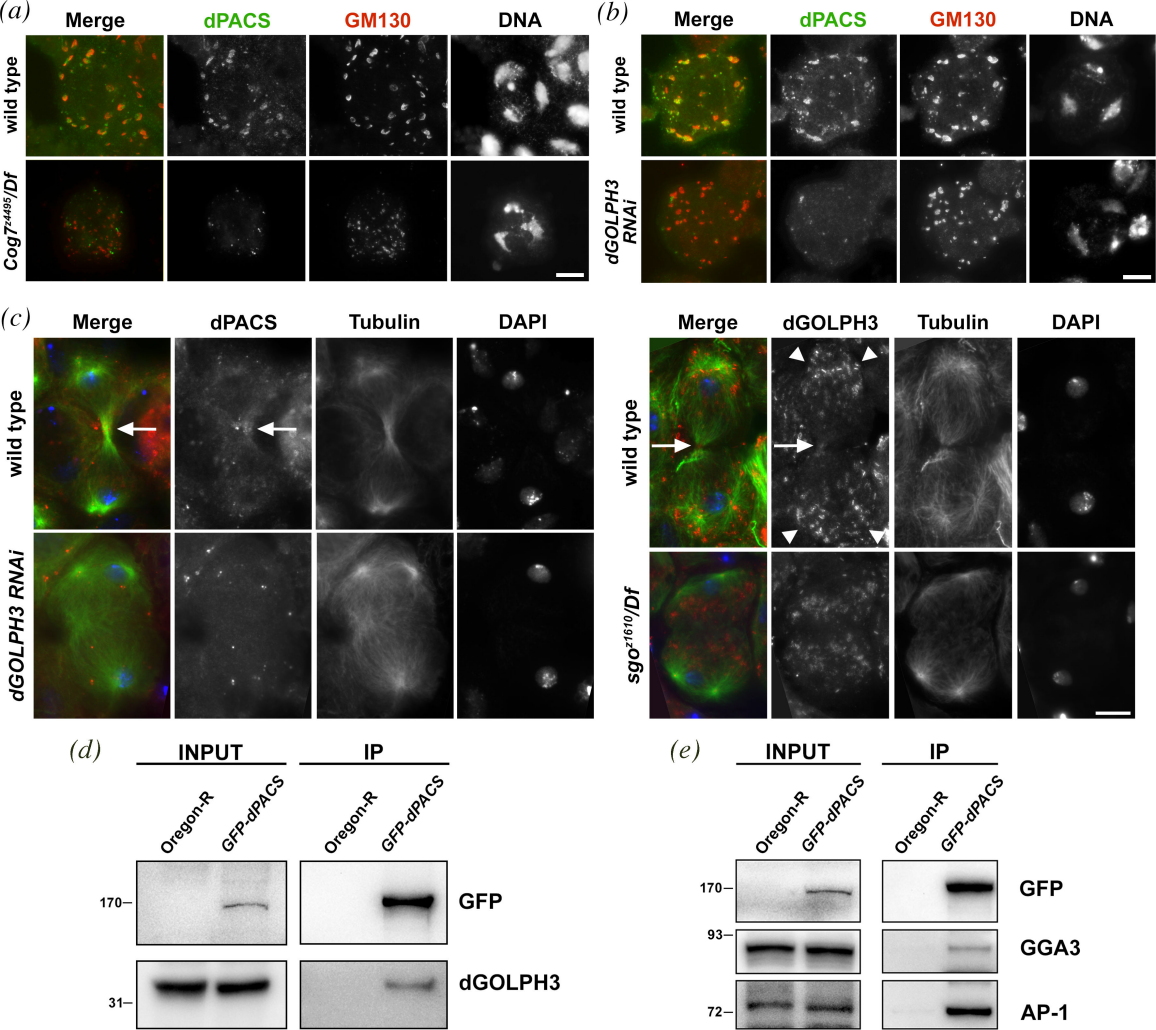
Relationship between dPACS, dGOLPH3 and Cog7 proteins. (*a*) Interphase spermatocytes from wild-type and *Cog7^z4495^/Df(3R)BSC861* (*Cog7^z4495^/Df*) mutants were stained for dPACS (green), GM130 (red) and DNA. (*b*) Interphase spermatocytes from wild-type and *Bam>GOLPH3RNAi (dGOLPH3RNAi*) testes were stained for dPACS (green), GM130 (red) and DNA. (*a,b*) *n* = 30 spermatocytes were examined per each genotype, randomly selected from images taken in five independent experiments. (*c*) Left panel: telophase spermatocytes from wild-type and *dGOLPH3RNAi* testes were stained for dPACS (red)*,* α-tubulin (*green*) and DNA (blue). Right panel: telophase spermatocytes from wild-type and *sgo^z1610^/Df* mutant testes were stained for dGOLPH3 (red)*,* α-tubulin (green) and DNA (blue). White arrows point respectively to dPACS and dGOLPH3 accumulation at the cleavage site; arrowheads point to dGOLPH3 signals at the cell poles. *n* = 25 mid- to late telophase spermatocytes were examined per each genotype, randomly selected from images taken in five independent experiments. (*a–c*) Scale bar: 10 μm. (*d*) GFP-dPACS was immunoprecipitated from GFP-dPACS transgenic adult heads with GFP trap and blotted with anti-dGOLPH3. Wild-type no-transgenic fly heads (Oregon-R) were used as negative control. Two per cent of the total lysate and the immunoprecipitates were loaded and probed with the indicated antibody. Molecular masses are in kilodaltons. (*e*) GFP-dPACS was immunoprecipitated from GFP-dPACS transgenic adult heads with GFP trap and probed for the indicated proteins. Wild-type no-transgenic fly heads (Oregon-R) were used as negative control. Two per cent of the total lysate and the immunoprecipitates were loaded and probed with the indicated antibody. (*d,e*) Co-IP experiments were performed in triplicate. Molecular masses are in kilodaltons.

Our previous characterization revealed that several other genes are essential for both Golgi structure maintenance and spermatocyte cytokinesis including *dGOLPH3* and *Cog7* [29,30,42,38]. To investigate the functional relationship between dPACS and Cog7, immunofluorescence analysis was used to examine the localization of dPACS in *Cog7^z4495^/Df(3R)BSC861* (*Cog7*) mutants and GFP-Cog7 in *sgo/Df* mutants (figure 7*a*). Upon loss of Cog7, dPACS protein failed to accumulate to the Golgi stacks and appeared localized in a few small puncta, some of which appear to localize in the proximity of Lva and GM130 in primary spermatocytes (figure 7*a*; electronic supplementary material, figure S7a). Similar immunofluorescence experiments showed that dGOLPH3 depletion driven by *Bam-Gal4* (*Bam>dGOLPH3*RNAi) impair dPACS localization in interphase spermatocytes (figure 7*b*; electronic supplementary material, figure S7b). Conversely, loss of dPACS did not affect the localization of either Cog7 or dGOLPH3 at the Golgi stacks in primary spermatocytes (electronic supplementary material, figure S7a,b). Next, we investigated the relationship between dPACS and AP-1 in male meiotic cells (electronic supplementary material, figure S6). In wild-type primary spermatocytes stained for AP-1 and the Golgi marker Rab1, AP-1 enrichment at the Golgi appeared slightly offset relative to Rab1 as expected for proteins on opposite sides of the Golgi stacks (electronic supplementary material, figure S6b). Conversely in *sgo^z1610^*/*Df* mutant spermatocytes, AP-1 was displaced from the Golgi stacks and localized in small puncta which appeared to be weakly associated with Rab1 (electronic supplementary material, figure S6b). These findings indicate that the dPACS function is required for the recruitment of AP-1 to the Golgi.

Next, we investigated the reciprocal dependence of dGOLPH3 and dPACS proteins in cells undergoing cytokinesis. Immunostaining for tubulin and dPACS revealed that dGOLPH3 function is required to localize dPACS at both the poles and the cleavage site of telophase spermatocytes (figure 7*c*). Similar to *dGOLPH3* mutants [29] telophase spermatocytes from *sgo/Df* mutants displayed a cytokinesis phenotype characterized by defective central spindles and failure to stabilize Pav (figure 2*c*), a direct molecular partner of dGOLPH3 [43]. Immunostaining for tubulin and dGOLPH3 revealed that all wild-type telophase spermatocytes displayed dGOLPH3 accumulation at the poles and at the cleavage site (figure 7*c*). Similar to wild-type, dGOLPH3 was concentrated at the cell poles in all *sgo/Df*telophase spermatocytes (figure 7*c*). However, consistent with the failure of Pav protein to localize to the spindle midzone, dGOLPH3 did not concentrate at the cell equator of dividing spermatocytes lacking dPACS (figure 7*c*).

Taken together, these data indicate that proper localization of dPACS at the Golgi organelles requires wild-type function of Cog7 and dGOLPH3 and that dPACS and dGOLPH3 proteins are mutually dependent for localization to the midzone during cytokinesis.

Our findings that dPACS and GOLPH3 are interdependent for their localization during cytokinesis, raise the question whether these proteins could physically interact. To test dPACS and dGOLPH3 interaction, we used co-immunoprecipitation (Co-IP) assay. GOLPH3 co-precipitated with dPACS in fly head extracts expressing GFP-dPACS (figure 7*d*). In agreement with previous work in mammalian cells [6,8], our Co-IP experiments also revealed the interaction of dPACS with AP-1 and GGA3 proteins (figure 7*e*).

### 2.4. *dPACS* mutants display defects in microtubule acetylation and severe bang sensitivity

In humans, variants of hPACS1 and hPACS2 proteins have been associated with epileptic encephalopathy and seizures [25–28]. A single recurrent *de novo* missense mutation in *hPACS1*, c607C>T (p.Arg203Trp; PACS1^R203W^), was identified in the majority of PACS1-NDD patients [25–27] (electronic supplementary material, figure S2). Recent work identified PACS1 as an *in vivo* effector of class IIb deacetylase HDAC6, which deacetylates α-tubulin to control the Golgi elements in dendrites [18]. It has been proposed that the PACS1^R203W^ substitution behaves as a gain of function mutation that increases the PACS1/HDAC6 interaction and potentiates deacetylase activity. As a consequence, the PACS1^R203W^ substitution increases the PACS1-dependent deacetylation of α-tubulin, causing Golgi fragmentation and disturbing microtubule organization in patient-derived cells and PACS1-NDD mice [18].

**Figure 8.**
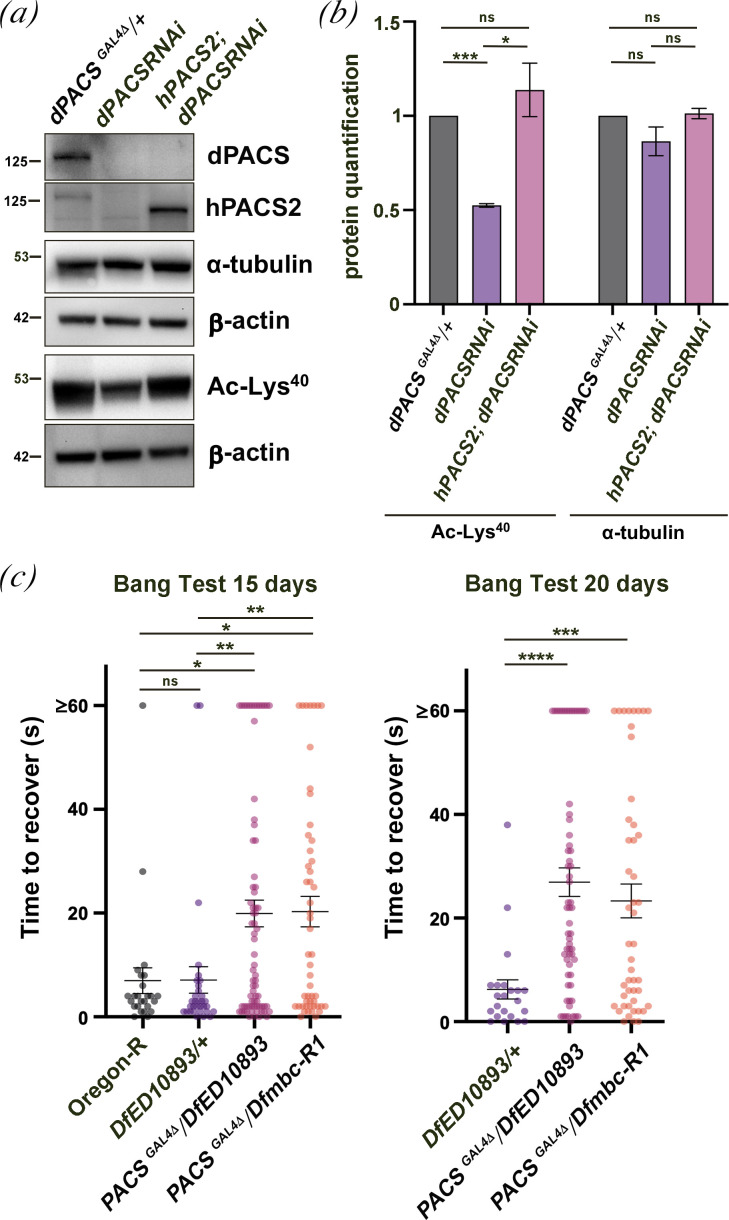
Loss of dPACS leads to defects in tubulin acetylation and severe bang sensitivity. (*a*) WB of total α-tubulin and Ac-Lys^40^-α-tubulin (Ac-Lys^40^) in protein extracts of adult heads from *dPACS^GAL4Δ^/+* flies (used as a control), flies expressing *UAS::dPACSRNAi* under the control of *dPACS^GAL4^ (dPACSRNAi),* and flies depleted of dPACS and simultaneously expressing hPACS2 (*hPACS2; dPACSRNAi*)*.* Flies were raised at 25°C. β-actin was used as a loading control. Molecular weights are in kilodaltons. (*b*) Quantification of the protein levels for α-tubulin and Ac-Lys^40^-α-tubulin (Ac-Lys^40^) in WB from animals of genotypes described in (*a*). Band intensities from three independent experiments were quantified using Image Lab software (Bio-Rad). The intensity of each band relative to the intensity of the loading control (β-actin) was normalized to the control (*dPACS^GAL4Δ^/+*). Data are mean ± s.d. *p < 0.05; ***p < 0.001 (unpaired Student’s *t*‐test). (*c*) *dPACS*-null flies (*dPACS^GAL4Δ^/Df(3R)ED10893* and *dPACS^GAL4Δ^/Df(3R)mbc-R1*) exhibit severe bang sensitivity. Oregon-R and heterozygous animals (*Df(3R)ED10893/+*) were used as a control. Data in the scatter plots are mean ± s.e.m. *p < 0.05; **p < 0.01; ***p < 0.001; ****p < 0.0001 (Mann–Whitney *U*-test).

To test whether dPACS functions as an *in vivo* regulator of α-tubulin acetylation in flies, we used WB analysis of total α-tubulin and Ac-Lys^40^ α-tubulin. These results indicated that the Ac-Lys^40^ α-tubulin levels were significantly reduced upon dPACS depletion in both fly testes and heads (electronic supplementary material, figure S8a; figure 8*a*,*b*). This result appears at odds with the slight increase in the level of Ac-Lys^40^ α-tubulin that was reported in WB from Pacs1^KO^ MEFs [18]. However, the loss of *PACS1* in Pacs1^KO^ MEFs was accompanied by an increase of PACS2 protein levels suggesting that the *PACS2* paralogue can mitigate the lack of *PACS1*. We tested whether the GAL4 that replaced *dPACS* coding sequence (*dPACS ^GAL4Δ^*) could drive the expression of the *UAS-hPACS2* transgene and rescue the reduced levels of Ac-Lys^40^ α-tubulin observed in fly heads depleted of dPACS. Additionally, because GAL4 is more active at 28°C than at 25°C, we could also express the *hPACS2* cDNA at two different temperatures. At 25°C, the expression of *hPACS2* cDNA driven by *dPACS ^GAL4Δ^* could rescue the reduction of Ac-Lys^40^ α-tubulin caused by dPACS knockdown, indicating conserved functions of the PACS proteins in an invertebrate model system (figure 8*a*,*b*). In contrast, at 28°C, expression of *hPACS2* failed to rescue the Ac-Lys^40^ α-tubulin defects (electronic supplementary material, figure S8b,c).

A characteristic phenotype resembling human epilepsy is bang sensitivity in flies [44]. Bang sensitivity measures the time required for flies to right themselves and move after a brief vortex in a vial. Interestingly *dPACS* null flies were severely bang sensitive when compared with control animals (figure 8*c*). This assay was first conducted on groups of 15-day-old flies of the selected genotypes, raised at 28°C and repeated on groups of 20-day-old flies in the same conditions. Wild-type flies righted themselves almost instantaneously after the mechanical stress (figure 8*c*). Conversely, *dPACS* null flies displayed uncoordinated limb movements for more than 20 s after the mechanical stress. Interestingly, the phenotypic defect of *dPACS* mutants increased in 20-day-old flies, suggesting a progressive age-dependent alteration of the nervous system (figure 8*c*).

## 3. Discussion

Studies in diverse model systems revealed that PACS family proteins regulate secretory and endocytic trafficking from invertebrates to humans [1,6–10,16,17,20,21].

In this paper, we have provided the first compelling demonstration that PACS function is required for cytokinesis. We demonstrate that the unique Drosophila homologue of human PACS1 and PACS2 proteins is essential for contractile ring constriction and vesicle trafficking in dividing cells. Loss of dPACS did not prevent the recruitment of Anillin and non-muscle MyoII at the cleavage furrow in spermatocytes and neuroblasts during the early stages of cytokinesis. However, the Anillin/MyoII rings assembled in dPACS mutants were defective and underwent minimal constriction during late telophase. This is consistent with our previous characterization of the *sgo ^z1610^* homozygotes which revealed defects in F-actin assembly and anillin rings constriction [31]. Failure to assemble functional actomyosin rings coupled with defective cleavage furrow ingression is a common phenotype in mutants affecting other proteins of the trafficking machinery in Drosophila spermatocytes [45,46]. More specifically these include important Golgi trafficking regulators such as the tethering COG subunits Cog5 and Cog7 [30,47], the PI(4)P effector GOLPH3 [29], the Rab1 GTPase [42], the TRAPP II component Brunelleschi [48], the vesicular coat COPI and COPII proteins [49,50], syntaxin 5 [51], the Golgi PI 4-kinase and membrane trafficking regulator Four wheel drive [52,53] and the ER–Golgi trafficking regulator Zw10 [40]. Additionally, analysis of mutants with early defects in the progression of the cleavage furrow in Drosophila spermatocytes, led to identify the Rab11 and Arf6 GTPases [54,55] and the exocyst proteins Exo84 and Sec8 [41] which regulate endocytic and recycling trafficking. Secretory and endocytic trafficking pathways have been implicated in actomyosin ring contraction in several other animal models [46,56,57]. In this context, defects in furrow ingression and contractile ring organization were observed in clathrin null cells of *Dictyostelium discoideum* where Myo II failed to form a functional ring, a phenotype that is comparable to that observed in *dPACS* mutant cells [58]. These observations have led to the proposal that during cytokinesis, actomyosin ring assembly and constriction are strictly coupled with vesicle trafficking and plasma membrane remodelling at the cleavage site [46,52,59,60]. Consistent with this model, live imaging of fluorescently tagged vesicles in Drosophila embryos showed that vesicles of endosomal origin and F-actin are targeted as a unit to the cleavage furrow, relying on central spindle microtubules [61].

Our findings that dPACS localizes at the Golgi organelles with GOLPH3 and Cog7, are in line with a role in Golgi trafficking. In agreement with this hypothesis, immunofluorescence analysis and TEM microscopy revealed altered architecture of Golgi organelles in interphase mutant spermatocytes indicating defective vesicular trafficking through these compartments. Consistent with previous work on hPACS1 [1,6], our findings indicate that dPACS interacts with the AP-1 adaptor protein and controls its recruitment to the Golgi stacks. On the other hand, proper localization of dPACS to the Golgi depends on wild-type functions of dGOLPH3 and Cog7 proteins. Furthermore, dPACS and dGOLPH3 form a complex and are mutually dependent for localization to the cleavage furrow. Taken together, our data indicate that dPACS, by associating with dGOLPH3 controls the flow of vesicle trafficking and contractile ring constriction during cytokinesis. Indeed, we have previously demonstrated that dGOLPH3 is a PI(4)P effector which exerts a key molecular function in coupling vesicle trafficking to actomyosin ring formation [29,43]. dGOLPH3 regulates the localization of PI(4)P-enriched secretory organelles at the cleavage furrow [29] and interacts with components of the vesicle trafficking and the cytokinesis machinery including MyoII and Pav [29,43]. Importantly, the reciprocal dependence between MyoII and PI(4)P-GOLPH3 controls centralspindlin stabilization at the invaginating membrane and contractile ring structure during cytokinesis [43]. In agreement with the lack of dGOLPH3 at the cleavage site, *dPACS* mutant spermatocytes displayed faint localization of the centralpindlin protein Pav and defects in central spindle stability.

We cannot exclude that the alterations in the central spindles of *dPACS* dividing spermatocytes may also result from the decrease in α-tubulin acetylation in mutant testes and the consequent effects on microtubule organization. There is much evidence that α-tubulin acetylation impacts microtubule structure and stability [62,63]. In this context, the defects in α-tubulin acetylation could contribute to male sterility of *dPACS* mutants by affecting the stability of flagellar microtubules. Indeed, tubulin acetylation is a conserved reversible modification of axonemal α-tubulin in several organisms including Drosophila and a key determinant of flagellar motility [64,65]. Recent work has shown that PACS1 binds class IIb deacetylase HDAC6, which deacetylates α-tubulin [18]. Moreover, the missense mutation PACS1^R203W^, identified in the majority of PACS1-NDD patients [25–27], potentiates PACS1/HDAC6 interaction leading to increased deacetylation of α-tubulin affects microtubule organization and Golgi structure in patient-derived cells and PACS1 syndrome mice [18]. In contrast with the slight increase of Ac-Lys^40^ α-tubulin that was reported in Pacs1^KO^ MEFs [18], our analysis showed that the Ac-Lys^40^ α-tubulin levels were significantly reduced upon dPACS depletion in both Drosophila testes and heads. However, the loss of *PACS1* in Pacs1^KO^ MEFs was coupled with an increased expression of PACS2 suggesting that the *PACS2* paralogue can compensate for the lack of *PACS1* potentially explaining this discrepancy. Importantly, expression of the *UAS-hPACS2* transgene at 25°C can rescue the reduced levels of Ac-Lys^40^ α-tubulin in fly heads depleted of dPACS, indicating conserved molecular functions of the PACS proteins in an invertebrate model system. Further investigation will be required to clarify whether the expression of *UAS-hPACS2* cDNA in flies might be toxic at a temperature higher than 25°C or might affect a Drosophila deacetylase.

In this context, it remains to be clarified whether dPACS interacts with HDAC6 as described for human PACS1 and how it regulates its activity. Our analysis, by showing that loss of dPACS perturbs Ac-Lys^40^ α-tubulin levels indicates that dPACS, like many HDAC6 interacting proteins [66–68] might negatively regulate HDAC6 or another deacetylase. However, our observations seem at odds with the effects of the missense mutation PACS^R203W^ in human cells which leads to increased HDAC6 activity and decreased acetylated tubulin [18]. Future analysis of null flies expressing human PACS1^R203W^ will shed light on the effects of the gain-of-function mutation that is common in most PACS1-NDD. Importantly, HDAC6 targets several sites in α-tubulin in addition to Lys^40^ [69]. A recent study in *D. melanogaster* has demonstrated that HDAC6 affects microtubule stability in synaptic terminals by regulating the deacetylation of a conserved α-tubulin Lys^394^ and not Lys^40^ [70]. Moreover, a mutation blocking Lys^394^ acetylation causes neuron type-specific effects when microtubule stability is altered [70]. Therefore, an in-depth analysis of the HDAC6/PACS interplay should take into consideration neuron-specific effects and multiple HDAC6 targets sites in α-tubulin.

The bang sensitivity in *dPACS* null flies closely parallels the epilepsy syndrome in PACS-NDD patients, providing a genetically tractable model for investigating novel strategies to treat the patients. Importantly, a bang-sensitive phenotype has been observed in mutants carrying a null allele in the fly *wdr37* which encodes the fly orthologues of human WDR37 (WD40 repeat-containing protein). WDR37, a member of the WD40 repeat protein family, is highly conserved in vertebrates and invertebrates, and has been reported to interact with PACS proteins in interactome-based studies performed in several model systems [71–74]. Additionally, in lymphocytes, PACS1 and WDR37 bind and stabilize each other and the WPACS1/WDR37 complex is required for maintaining the peripheral lymphocyte population [74]. The intermolecular dependency of PACS1 and WDR37 has also been reported in *C. elegans,* suggesting that it is evolutionarily conserved [5]. Recent work revealed strong similarities between PACS1-NDD, PACS2 syndrome and the recently identified *WDR37* syndromic neurological disorder [75,76]. Further studies will be needed to clarify the functional relationship between fly *wdr37* and *dPACS* genes, which may contribute to understanding the cellular and molecular mechanisms underlying the PACS/WDR37 syndromes.

The treatment of the PACS1 syndrome mice with antisense oligonucleotides targeting the PACS1^R203W^ variant could restore the neuronal structure or communication suggesting that PACS1-NDD may be treated with targeted therapies [18]. Because one challenge for antisense oligonucleotide therapies is their inability to cross the blood–brain barrier, one proposed alternative is the use of inhibitors of HDAC6 or targeting other PACS1-client proteins [18,75].

Our characterization of *dPACS* null mutants has revealed Golgi structural defects, cytokinesis failures and reduced levels of Ac-Lys^40^ α-tubulin which may question the use of inhibitors of HDAC6 to treat PACS1-NDD. The superior genetic tools available in *D. melanogaster* and the presence of a unique homologue of PACS proteins will offer unique opportunities to further dissect the underlying molecular mechanisms of the PACS-related NDD and to perform therapeutic screening.

## 4. Methods

### 4.1. Fly stocks and transgenes

Drosophila strains were reared in standard cornmeal-agar medium (#789211, NutriFly BF, Genesee Scientific) and maintained at 25°C unless otherwise specified. Wild-type Oregon-R (Or-R) flies were used as a reference unless otherwise noted. The RNAi experiments were performed at 28°C. *UAS-dGOLPH3 RNAi* were obtained from the Vienna Drosophila Resource Center Collection (VDRC, #46150 [77]) and were described previously [29]. The following fly strains were from the Bloomington Drosophila Stock Center (BDSC, Indiana University, Bloomington, IN, USA): *UAS-GFP* (#4775), *P{EPgy2}KrT95D^EY1255^* (#20754 [78]), *Mi{Trojan-GAL4.2}KrT95D^MI00927-TG4.2^* (#78873 [79], carrying a Minos-mediated integration cassette insertion resulting in the replacement of the entire coding sequence of *KrT95D* with GAL4 (*KrT95D^GAL4Δ^*), *P{TRiP.HMC05743}attP40* (#64870 [80]), *Df(3R)ED10893* (#28827 [81]), *Df(3R)Exel6196* (#7675 [82]), *Df(3R)BSC861* (# 29984 [83]), *PBac{UAS-hPACS2.B}VK00037* (#92022) and TI{TI}Rab4^EYFP^ (#6542 [84]). Transgenic *bam-Gal4* was used to deplete either dGOLPH3 or dPACS in fly testes [85]. Flies expressing GFP-Cog7 were described previously [30]. *Cog7^z4495^* and *sgo^z1610^* mutant strains were described previously [30,31,38] and were from the C. Zuker collection of male sterile mutants [31,86].

### 4.2. Molecular biology and rescue experiments

DNA sequencing of dPACS protein coding exons was performed from individuals carrying the *sgo^z1610^* mutation and the original Zuker-background chromosome. To generate the GFP-dPACS construct, the EGFP coding sequence was fused in frame to the amino terminus of the full-length *CG5405* cDNA and cloned into the pCaSpeR4 under the control of α1-tubulin promoter as described previously [29,41]. Transgenic flies were generated by P-element-mediated germline transformation, performed by Bestgene Inc. (Chino Hills, CA, USA). *GFP-dPACS* was crossed into the *sgo^z1610^*/*Df(3R)ED10893* mutant background to test for phenotypic rescue of male meiotic cytokinesis failures. To obtain a GST fusion to dPACS protein, the cDNA corresponding to the 547−1175 amino acid fragment of Drosophila dPACS was cloned into a pGEX-6p-2 (GE Healthcare), as described previously [29].

### 4.3. Generation of anti-dPACS antibodies

Polyclonal antibodies were generated against the 547−1175 amino acid fragment of dPACS. The GST-tagged polypeptide was expressed in BL21-CodonPlus (DE3) cells (Invitrogen) and purified using HiTrap affinity columns (GTtrap FF, and GSTtrap HP columns, GE Healthcare) operated with AKTA 900 fast protein liquid chromatography. Polyclonal antisera against the purified GST-protein were produced in rabbits and mice (Agro-Bio Services; https://www.agro-bio.com/). The anti-GST-dPACS antisera were first depleted of anti-GST antibodies and then affinity-purified against GST-dPACS.

### 4.4. Protein extracts and Western blotting

Protein extracts of adult testes and adult heads were performed as described previously [87]. Briefly, 100 testes or 50 heads from males of each genotype were homogenized in 100 µl of lysis buffer (25 mM Tris–HCl pH 7.4; 150  mM NaCl, 1  mM EDTA; glycerol 5%; 1% NP40) containing protease inhibitor (#11697498001, Roche) for 30−40 min on ice. All lysates were clarified two times by centrifugation at 12 000 ×*g* for 10 min. Protein concentration was quantified using the Pierce BCA Protein Assay Kit (#233225, Thermo Scientific). Samples were separated on Mini-protean TGX Stain-Free Gel gels (Bio-Rad Laboratories) and blotted to PVDF membranes (#1620177, Bio-Rad) using the Trans-Blot Turbo TM Transfer System (#1704150, Bio-Rad). Membranes were blocked in EveryBlot Blocking Buffer (#12010020, Bio-Rad), followed by incubation with primary and secondary antibodies diluted in TBS-T (20 mM Tris-HCl pH 7.5, 150 mM NaCl, 0.05% Tween 20). Primary antibodies were as follows: mouse anti-dGOLPH3 (#S11047/1/56; 1:2500 [29]); rabbit anti-dGOLPH3 (#G49139/77; 1:5000 [29]); rabbit anti-dPACS (#L13067b/3032119/77, this study; 1:2000); mouse anti-dPACS (#S13067b/3/35, this study; 1:1000); rabbit anti-β-actin (#4967, Cell signaling Technology; 1:500); rabbit anti-GFP (#TP401, Torrey Pines; 1:1000); rabbit anti-Drosophila AP-1 γ (a gift from Prof. M. S. Robinson, University of Cambridge; 1:2000 [88]); rabbit anti-GGA3 (a gift from Prof. M. S. Robinson, University of Cambridge; 1:2000 [88]); mouse anti-α-tubulin (#T6199, Sigma Aldrich); and mouse anti-acetyl-α-tubulin (Lys 40, #T7451, Sigma Aldrich; 1:200). HRP-conjugated secondary antibodies, used at 1:5000, were as follows: goat anti-rabbit IgG (H+L) (#170−6515, Bio-Rad; 1:5000) and goat anti-mouse IgG (H+L) (#170−6516, Bio-Rad; 1:5000). After incubation with the antibodies, blots were washed (3 × 5 min) in TBS-T. Blots were then incubated five min with ECL substrate (#1705062 and #1705060, Bio-Rad) and the HRP-ECL signals were revealed using the ChemiDocTM XRS gel imaging system (Bio-Rad). Band intensity quantification was performed using the gel analyser tool in Image Lab (Bio-Rad) and Fiji/ImageJ software [89].

### 4.5. Co-immunoprecipitation analysis

Co-IP experiments were carried out following the protocol that was previously described in [87]. Co-IP from either GFP-dPACS or OR-R samples were performed using GFP trap-A purchased from Proteintech (#gta-20). All preparation steps for Co-IP were carried out on ice and both control agarose beads (#bab-20, Proteintech) and GFP trap-A beads were washed three times before use, with 1 ml of lysis buffer (25 mM Tris–HCl, pH 7.4; 150  mM NaCl, 1  mM EDTA; glycerol 5%; 1% NP40) containing protease inhibitor (#11697498001, Roche). After each washing step, the beads were centrifuged at 2500 ×*g* for two min at 4°C. Two hundred and fifty heads from Oregon-R or flies expressing GFP-dPACS, were lysed in 500 µl of lysis buffer and precleared by incubation for 1 h on wheel at 4°C with control agarose beads. Four per cent of each lysate was retained as the ‘input’ and the remainder was incubated with 20 μl of GFP trap-A beads for 2 h at 4°C. The beads were washed four to five times with 1 ml lysis buffer by inverting the tubes. After the final wash, GFP-dPACS and its molecular interactors were eluted by boiling in 30 μl of 2× SDS sample buffer (20% glycerol, 4% SDS, 0.2% BBF, 100 mM Tris–HCl (pH 6.8), 200 mM DTT). Co-IP experiments were performed in triplicate.

### 4.6. Microscopy and histology

Multinucleate spermatids were quantified in squashed preparations of live adult testes from 1- to 3-day-old males, dissected as described in [31]. Images of testis preparations were collected using a charged-coupled device (CCD camera, Qimaging QICAM Mono Fast 1394 Cooled), connected to a Nikon Axioplan epifluorescence microscope equipped with a 40× phase contrast objective.

To visualize mitotic chromosomes, larval brains were dissected in NaCl 0.7%, treated with hypotonic solution for seven min and fixed in 45% acetic acid as described in [30].

Immunofluorescence analysis of primary spermatocytes or neuroblasts was made with testes or brains from pupae. To visualize tubulin and either myosin, anillin, Pav or Lva, preparations were fixed using 3.7% formaldehyde in PBS and then squashed in 60% acetic acid, as previously described in [90]. For other immunostaining of spermatocytes, testes were dissected in PBS and then transferred into 5 µl of 4% methanol-free formaldehyde (#18814-20, Polysciences) in PBS on a 20 × 20 coverslip. After one min, samples were gently squashed, left for six min at room temperature and then immersed into liquid nitrogen. After coverslip removal with a razor blade, preparations were rinsed 2 × 5 min in PBS. Samples were permeabilized (2 × 10 min) in PBS with 0.1% Triton X-100 (PBT) and then blocked with 3% BSA in PBT for 30 min. Immunofluorescence analysis of pupal neuroblasts was carried out according to [90].

For whole-mount immunostaining of larval brains, we used the following protocol. Briefly, brains from *UAS-GFP; KrT95D^GAL4^*^/^*^+^* third instar larvae were dissected in PBS, collected into a microcentrifuge tube and fixed in 4% methanol-free formaldehyde in PBT for 1 h on ice. Samples were permeabilized in PBT (3 × 10 min) and blocked in PBT containing 5% BSA for 30 min before immunostaining.

Monoclonal antibodies were used to stain α-Tubulin (#T6199, 1: 300, Sigma Aldrich and #F2168, 1:200, Sigma-Aldrich Anti-α-Tubulin FITC-conjugated antibody) and Elav (#Elav-9F8A9, 1:100, Developmental studies hybridoma bank). Polyclonal antibodies were as follows: mouse anti-dPACS (#S13067b/3/35, this study; 1:500); rabbit anti-Asterless (Asl, gift of C. Gonzalez, IRB-Barcelona, Spain; 1:500 [91]); rabbit anti-Pav (gift of D.M. Glover, California Institute of Technology, Pasadena, California; 1:100 [36]); rabbit anti-Zip (gift of R. Karess, Paris Diderot University, France; 1:500 [35]); rabbit anti-Lava lamp (gift from O. Papoulas, University of Texas at Austin; 1:300 [37]), rabbit anti-GM130 (#ab30637, Abcam; 1:200); rabbit anti-anillin antibody (Y0136L/4082307/77; 1:1000 [41]); rabbit anti-GFP (#TP401, Torrey Pines; 1:1000); rabbit anti-AP-1 γ (a gift from Prof. J. Hirst and Prof M. S. Robinson, University of Cambridge; 1:2000 [88]); mouse anti-Rab1 antibody S12085a (1: 750 [42]); rabbit anti-Rab1 L12085a/169 (1:1000 [42]). Secondary antibodies were: Alexa 555-conjugated goat anti-rabbit IgG (#A21430, Life Technologies; 1:300); Alexa 488-conjugated anti-mouse IgG (Jackson ImmunoResearch; 1:50); Alexa 488-conjugated anti-rabbit IgG (Jackson ImmunoResearch; 1:800). Samples were incubated with primary antibodies, diluted in PBT containing 3% BSA, overnight at 4°C and with secondary antibodies for 1 h at room temperature. After immunostaining, samples were rinsed in PBS and mounted in Vectashield mounting medium with DAPI (#H-1800, Vector Laboratories).

Fluorescence images from fixed spermatocytes were acquired using a Zeiss Axio Observer Z1 (Carl Zeiss) equipped with a 100×/1.3 NA oil immersion objective and a charged-coupled device (Axiocam 503 mono CCD camera) and an HXP 120 V inclusive built-in power supply, lamp module. Images were processed using Zeiss ZEN2 software and Adobe Photoshop. The Golgi compartment was demarcated using the freehand selection tool and the area was measured using the analyse tool of Fiji/ImageJ software.

### 4.7. Transmission electron microscopy

Testes from both wild-type and *sgo/Df* mutant pupae were dissected in phosphate-buffered saline (PBS) and fixed overnight at 4°C in 2.5% glutaraldehyde in PBS. After rinsing in PBS (3 × 15 min), the samples were post-fixed in 1% osmium tetroxide in PBS for 1 h at 4°C. Preparations were then washed for 30 min in distilled water, dehydrated through a series of graded ethyl alcohols and then embedded in an Epon-Araldite mixture and polymerized at 60°C for 48 h. Ultrathin sections were cut with a LKB Ultratome NOVA, equipped with a diamond knife. The sections were collected with copper slot grids coated with formvar. The sections were stained with 2% aqueous uranyl acetate for 20 min in the dark and then with lead citrate for two min. The preparations were observed with a Tecnai G2 Spirit EM (FEI Eindhoven, The Netherlands) equipped with a Morada CCD camera (Olympus, Tokyo, Japan).

### 4.8. Bang sensitivity assays

Fifteen-day-old or 20-day-old flies, raised at 28°C, were selected in groups of five and transferred into new vials the day before the assay, to recover from CO_2_ anaesthesia. Prior to testing, flies were transferred into empty vials. To assess seizure, vials were vortexed at maximum speed (VORTEX Genius 3, IKA cat. no. 3340000) for 30 s, as described in [76]. Seizure duration was measured as the time required to regain posture and start climbing the wall for each fly. At least 25 flies were tested per trial per each genotype.

### 4.9. Statistical analysis

The statistical analysis was performed using Prism 8 (GraphPad Prism, USA). Statistical parameters (value of *n*, mean, s.d., s.e., s.e.m., *p*-value) and statistical tests used in each experiment are reported in the corresponding figure legend.

## Data Availability

Supplementary material is available online [92].

## References

[1] Wan L, Molloy SS, Thomas L, Liu G, Xiang Y, Rybak SL, Thomas G. 1998 PACS-1 defines a novel gene family of cytosolic sorting proteins required for trans-Golgi network localization. Cell **94**, 205–216. (10.1016/s0092-8674(00)81420-8) 9695949

[2] Thomas G. 2002 Furin at the cutting edge: from protein traffic to embryogenesis and disease. Nat. Rev. Mol. Cell Biol. **3**, 753–766. (10.1038/nrm934) 12360192 PMC1964754

[3] Thomas G, Aslan JE, Thomas L, Shinde P, Shinde U, Simmen T. 2017 Caught in the act: protein adaptation and the expanding roles of the PACS proteins in tissue homeostasis and disease. J. Cell. Sci. **130**, 1865–1876. (10.1242/jcs.199463) 28476937 PMC5482974

[4] Youker RT, Shinde U, Day R, Thomas G. 2009 At the crossroads of homoeostasis and disease: roles of the PACS proteins in membrane traffic and apoptosis. Biochem. J. **421**, 1–15. (10.1042/bj20081016) 19505291 PMC4303049

[5] Byrd DT, Han ZC, Piggott CA, Jin Y. 2024 PACS-1 variant protein is aberrantly localized in Caenorhabditis elegans model of PACS1/PACS2 syndromes. Genetics **228**, e118. (10.1093/genetics/iyae118) PMC11457933 39031646

[6] Crump CM, Xiang Y, Thomas L, Gu F, Austin C, Tooze SA, Thomas G. 2001 PACS-1 binding to adaptors is required for acidic cluster motif-mediated protein traffic. EMBO J. **20**, 2191–2201. (10.1093/emboj/20.9.2191) 11331585 PMC125242

[7] Köttgen M *et al*. 2005 Trafficking of TRPP2 by PACS proteins represents a novel mechanism of ion channel regulation. EMBO J. **24**, 705–716. (10.1038/sj.emboj.7600566) 15692563 PMC549624

[8] Scott GK, Fei H, Thomas L, Medigeshi GR, Thomas G. 2006 A PACS-1, GGA3 and CK2 complex regulates CI-MPR trafficking. EMBO J. **25**, 4423–4435. (10.1038/sj.emboj.7601336) 16977309 PMC1589982

[9] Atkins KM, Thomas L, Youker RT, Harriff MJ, Pissani F, You H, Thomas G. 2008 HIV-1 Nef binds PACS-2 to assemble a multikinase cascade that triggers major histocompatibility complex Class I (MHC-I) down-regulation: analysis using short interfering RNA and knock-out mice. J. Biol. Chem. **283**, 11772–11784. (10.1074/jbc.m707572200) 18296443 PMC2431057

[10] Feliciangeli SF, Thomas L, Scott GK, Subbian E, Hung CH, Molloy SS, Jean F, Shinde U, Thomas G. 2006 Identification of a pH sensor in the furin propeptide that regulates enzyme activation. J. Biol. Chem. **281**, 16108–16116. (10.1074/jbc.m600760200) 16601116 PMC4293020

[11] Hinners I, Wendler F, Fei H, Thomas L, Thomas G, Tooze SA. 2003 AP‐1 recruitment to VAMP4 is modulated by phosphorylation‐dependent binding of PACS‐1. EMBO Rep. **4**, 1182–1189. (10.1038/sj.embor.7400018) 14608369 PMC1326413

[12] Schmidt V, Sporbert A, Rohe M, Reimer T, Rehm A, Andersen OM, Willnow TE. 2007 SorLA/LR11 regulates processing of amyloid precursor protein via interaction with adaptors GGA and PACS-1. J. Biol. Chem. **282**, 32956–32964. (10.1074/jbc.m705073200) 17855360

[13] Burgert T, Schmidt V, Caglayan S, Lin F, Füchtbauer A, Füchtbauer EM, Nykjaer A, Carlo AS, Willnow TE. 2013 SORLA-dependent and -independent functions for PACS1 in control of amyloidogenic processes. Mol. Cell. Biol. **33**, 4308–4320. (10.1128/mcb.00628-13) 24001769 PMC3811889

[14] Piguet V, Wan L, Borel C, Mangasarian A, Demaurex N, Thomas G, Trono D. 2000 HIV-1 Nef protein binds to the cellular protein PACS-1 to downregulate class I major histocompatibility complexes. Nat. Cell Biol. **2**, 163–167. (10.1038/35004038) 10707087 PMC1475706

[15] Crump CM, Hung CH, Thomas L, Wan L, Thomas G. 2003 Role of PACS-1 in trafficking of human cytomegalovirus glycoprotein B and virus production. J. Virol. **77**, 11105–11113. (10.1128/jvi.77.20.11105-11113.2003) 14512558 PMC224974

[16] Schermer B *et al*. 2005 Phosphorylation by casein kinase 2 induces PACS-1 binding of nephrocystin and targeting to cilia. EMBO J. **24**, 4415–4424. (10.1038/sj.emboj.7600885) 16308564 PMC1356326

[17] Jenkins PM, Zhang L, Thomas G, Martens JR. 2009 PACS-1 mediates phosphorylation-dependent ciliary trafficking of the cyclic-nucleotide-gated channel in olfactory sensory neurons. J. Neurosci. **29**, 10541–10551. (10.1523/jneurosci.1590-09.2009) 19710307 PMC2749268

[18] Villar-Pazos S *et al*. 2023 Neural deficits in a mouse model of PACS1 syndrome are corrected with PACS1- or HDAC6-targeting therapy. Nat. Commun. **14**, 6547. (10.1038/s41467-023-42176-8) 37848409 PMC10582149

[19] Myhill N, Lynes EM, Nanji JA, Blagoveshchenskaya AD, Fei H, Carmine Simmen K, Cooper TJ, Thomas G, Simmen T. 2008 The subcellular distribution of Calnexin is mediated by PACS-2. Mol. Biol. Cell **19**, 2777–2788. (10.1091/mbc.e07-10-0995) 18417615 PMC2441662

[20] Sieburth D *et al*. 2005 Systematic analysis of genes required for synapse structure and function. Nature **436**, 510–517. (10.1038/nature03809) 16049479

[21] Dombernowsky SL *et al*. 2015 The sorting protein PACS-2 promotes ErbB signalling by regulating recycling of the metalloproteinase ADAM17. Nat. Commun. **6**, 7518. (10.1038/ncomms8518) 26108729 PMC4481878

[22] Simmen T *et al*. 2005 PACS-2 controls endoplasmic reticulum–mitochondria communication and Bid-mediated apoptosis. EMBO J. **24**, 717–729. (10.1038/sj.emboj.7600559) 15692567 PMC549619

[23] Aslan JE *et al*. 2009 Akt and 14-3-3 control a PACS-2 homeostatic switch that integrates membrane traffic with TRAIL-induced apoptosis. Mol. Cell **34**, 497–509. (10.1016/j.molcel.2009.04.011) 19481529 PMC2744858

[24] Wheeler E *et al*. 2013 Genome-wide SNP and CNV analysis identifies common and low-frequency variants associated with severe early-onset obesity. Nat. Genet. **45**, 513–517. (10.1038/ng.2607) 23563609 PMC4106235

[25] Schuurs-Hoeijmakers JHM *et al*. 2012 Recurrent de novo mutations cause defective cranial-neural-crest migration and define a recognizable intellectual-disability syndrome. Am. J. Hum. Genet. **91**, 1122–1127. (10.1016/j.ajhg.2012.10.013) 23159249 PMC3516611

[26] Schuurs-Hoeijmakers J. 2016 Clinical delineation of the PACS1-related syndrome: report on 19 patients. Am. J. Med. Genet. Part **170**, 670–675. (10.1002/ajmg.a.37476) 26842493

[27] Stern D *et al*. 2017 Association of the missense variant p.Arg203Trp in PACS1 as a cause of intellectual disability and seizures. Clin. Genet. **92**, 221–223. (10.1111/cge.12956) 28111752 PMC5513756

[28] Olson HE *et al*. 2018 A recurrent de novo PACS2 heterozygous missense variant causes neonatal-onset developmental epileptic encephalopathy, facial dysmorphism, and cerebellar dysgenesis. Am. J. Hum. Genet. **102**, 995–1007. (10.1016/j.ajhg.2018.03.005) 29656858 PMC5986694

[29] Sechi S, Colotti G, Belloni G, Mattei V, Frappaolo A, Raffa GD, Fuller MT, Giansanti MG. 2014 GOLPH3 is essential for contractile ring formation and Rab11 localization to the cleavage site during cytokinesis in Drosophila melanogaster. PLoS Genet. **10**, e1004305. (10.1371/journal.pgen.1004305) 24786584 PMC4006750

[30] Belloni G, Sechi S, Riparbelli MG, Fuller MT, Callaini G, Giansanti MG. 2012 Mutations in Cog7 affect Golgi structure, meiotic cytokinesis and sperm development during Drosophila spermatogenesis. J. Cell Sci. **125**, 5441–5452. (10.1242/jcs.108878) 22946051 PMC7523631

[31] Giansanti MG, Farkas RM, Bonaccorsi S, Lindsley DL, Wakimoto BT, Fuller MT, Gatti M. 2004 Genetic dissection of meiotic cytokinesis in Drosophila males. Mol. Biol. Cell **15**, 2509–2522. (10.1091/mbc.e03-08-0603) 15004238 PMC404041

[32] Hu Y, Flockhart I, Vinayagam A, Bergwitz C, Berger B, Perrimon N, Mohr SE. 2011 An integrative approach to ortholog prediction for disease-focused and other functional studies. BMC Bioinform. **12**, 357. (10.1186/1471-2105-12-357) PMC3179972 21880147

[33] Di Tommaso P, Moretti S, Xenarios I, Orobitg M, Montanyola A, Chang JM, Taly JF, Notredame C. 2011 T-Coffee: a web server for the multiple sequence alignment of protein and RNA sequences using structural information and homology extension. Nucleic Acids Res. **39**, W13–W17. (10.1093/nar/gkr245) 21558174 PMC3125728

[34] Chintapalli VR, Wang J, Dow JAT. 2007 Using FlyAtlas to identify better Drosophila melanogaster models of human disease. Nat. Genet. **39**, 715–720. (10.1038/ng2049) 17534367

[35] Royou A, Sullivan W, Karess R. 2002 Cortical recruitment of nonmuscle myosin II in early syncytial Drosophila embryos: its role in nuclear axial expansion and its regulation by Cdc2 activity.. J. Cell Biol. **158**, 127–137. (10.1083/jcb.200203148) 12105185 PMC2173028

[36] Adams RR, Tavares AAM, Salzberg A, Bellen HJ, Glover DM. 1998 pavarotti encodes a kinesin-like protein required to organize the central spindle and contractile ring for cytokinesis. Genes Dev. **12**, 1483–1494. (10.1101/gad.12.10.1483) 9585508 PMC316841

[37] Sisson JC, Field C, Ventura R, Royou A, Sullivan W. 2000 Lava Lamp, a novel peripheral Golgi protein, is required for Drosophila melanogaster cellularization. J. Cell Biol. **151**, 905–918. (10.1083/jcb.151.4.905) 11076973 PMC2169433

[38] Frappaolo A *et al*. 2017 COG7 deficiency in Drosophila generates multifaceted developmental, behavioral and protein glycosylation phenotypes. J. Cell. Sci. **130**, 3637–3649. (10.1242/jcs.209049) 28883096 PMC5702061

[39] Hirst J, Carmichael J. 2011 A potential role for the clathrin adaptor GGA in Drosophila spermatogenesis. BMC Cell Biol. **12**, 22. (10.1186/1471-2121-12-22) 21599933 PMC3127973

[40] Wainman A, Giansanti MG, Goldberg ML, Gatti M. 2012 The Drosophila RZZ complex: roles in membrane traffic and cytokinesis. J. Cell Sci. **125**, 4014–4025. (10.1242/jcs.099820) 22685323 PMC3482314

[41] Giansanti MG *et al*. 2015 Exocyst-dependent membrane addition is required for anaphase cell elongation and cytokinesis in Drosophila. PLOS Genet. **11**, e1005632. (10.1371/journal.pgen.1005632) 26528720 PMC4631508

[42] Sechi S, Frappaolo A, Fraschini R, Capalbo L, Gottardo M, Belloni G, Glover DM, Wainman A, Giansanti MG. 2017 Rab1 interacts with GOLPH3 and controls Golgi structure and contractile ring constriction during cytokinesis in Drosophila melanogaster. Open Biol. **7**, 160257. (10.1098/rsob.160257) 28100664 PMC5303273

[43] Sechi S, Frappaolo A, Karimpour-Ghahnavieh A, Fraschini R, Giansanti MG. 2020 A novel coordinated function of Myosin II with GOLPH3 controls centralspindlin localization during cytokinesis in Drosophila. J. Cell. Sci. **133**, jcs252965. (10.1242/jcs.252965) 33037125

[44] Parker L, Howlett IC, Rusan ZM, Tanouye MA. 2011 Seizure and epilepsy: studies of seizure disorders in Drosophila. Int. Rev. Neurobiol ***99***, 1–21. (10.1016/b978-0-12-387003-2.00001-x) 21906534 PMC3532860

[45] Frappaolo A, Piergentili R, Giansanti MG. 2022 Microtubule and actin cytoskeletal dynamics in male meiotic cells of Drosophila melanogaster. Cells **11**, 695. (10.3390/cells11040695) 35203341 PMC8870657

[46] D’Avino PP, Giansanti MG, Petronczki M. 2015 Cytokinesis in animal cells. Cold Spring Harb. Perspect. Biol. **7**, a015834. (10.1101/cshperspect.a015834) 25680833 PMC4382743

[47] Farkas RM, Giansanti MG, Gatti M, Fuller MT. 2003 The Drosophila Cog5 homologue is required for cytokinesis, cell elongation, and assembly of specialized Golgi architecture during spermatogenesis. Mol. Biol. Cell **14**, 190–200. (10.1091/mbc.e02-06-0343) 12529436 PMC140237

[48] Robinett CC, Giansanti MG, Gatti M, Fuller MT. 2009 TRAPPII is required for cleavage furrow ingression and localization of Rab11 in dividing male meiotic cells of Drosophila. J. Cell Sci. **122**, 4526–4534. (10.1242/jcs.054536) 19934220 PMC2787463

[49] Kitazawa D, Yamaguchi M, Mori H, Inoue YH. 2012 COPI-mediated membrane trafficking is required for cytokinesis in Drosophila male meiotic divisions. J. Cell Sci. **125**, 3649–3660. (10.1242/jcs.103317) 22553212

[50] Matsuura Y, Kaizuka K, Inoue YH. 2024 Essential role of COPII proteins in maintaining the contractile ring anchoring to the plasma membrane during cytokinesis in Drosophila male meiosis. Int. J. Mol. Sci. **25**, 4526. (10.3390/ijms25084526) 38674111 PMC11050551

[51] Xu H, Brill JA, Hsien J, McBride R, Boulianne GL, Trimble WS. 2002 Syntaxin 5 is required for cytokinesis and spermatid differentiation in Drosophila. Dev. Biol. **251**, 294–306. (10.1006/dbio.2002.0830) 12435359

[52] Brill JA, Hime GR, Scharer-Schuksz M, Fuller MT. 2000 A phospholipid kinase regulates actin organization and intercellular bridge formation during germline cytokinesis. Development **127**, 3855–3864. (10.1242/dev.127.17.3855) 10934029

[53] Polevoy G, Wei HC, Wong R, Szentpetery Z, Kim YJ, Goldbach P, Steinbach SK, Balla T, Brill JA. 2009 Dual roles for the Drosophila PI 4-kinase Four wheel drive in localizing Rab11 during cytokinesis. J. Cell Biol. **187**, 847–858. (10.1083/jcb.200908107) 19995935 PMC2806325

[54] Giansanti MG, Belloni G, Gatti M. 2007 Rab11 Is required for membrane trafficking and actomyosin ring constriction in meiotic cytokinesis of Drosophila males. Mol. Biol. Cell **18**, 5034–5047. (10.1091/mbc.e07-05-0415) 17914057 PMC2096611

[55] Dyer N, Rebollo E, Domínguez P, Elkhatib N, Chavrier P, Daviet L, González C, González-Gaitán M. 2007 Spermatocyte cytokinesis requires rapid membrane addition mediated by ARF6 on central spindle recycling endosomes. Development **134**, 4437–4447. (10.1242/dev.010983) 18039970

[56] Burgess RW, Deitcher DL, Schwarz TL. 1997 The synaptic protein Syntaxin1 is required for cellularization of Drosophila embryos. J. Cell Biol. **138**, 861–875. (10.1083/jcb.138.4.861) 9265652 PMC2138053

[57] Frémont S, Echard A. 2018 Membrane Traffic in the Late Steps of Cytokinesis. Curr. Biol. **28**, R458–R470. (10.1016/j.cub.2018.01.019) 29689230

[58] Gerald NJ, Damer CK, O’Halloran TJ, De Lozanne A. 2001 Cytokinesis failure in clathrin-minus cells is caused by cleavage furrow instability. Cell Motil. Cytoskelet. **48**, 213–223. (10.1002/1097-0169(200103)48:33.0.co;2-v) 11223952

[59] Giansanti MG, Bonaccorsi S, Kurek R, Farkas RM, Dimitri P, Fuller MT, Gatti M. 2006 The Class I PITP Giotto Is Required for Drosophila Cytokinesis. Curr. Biol. **16**, 195–201. (10.1016/j.cub.2005.12.011) 16431372

[60] Giansanti MG, Fuller MT. 2012 What Drosophila spermatocytes tell us about the mechanisms underlying cytokinesis. Cytoskeleton **69**, 869–881. (10.1002/cm.21063) 22927345 PMC4165571

[61] Albertson R, Cao J, Hsieh T shih, Sullivan W. 2008 Vesicles and actin are targeted to the cleavage furrow via furrow microtubules and the central spindle. J. Cell Biol. **181**, 777–790. (10.1083/jcb.200803096) 18504302 PMC2396810

[62] Janke C, Montagnac G. 2017 Causes and consequences of microtubule acetylation. Curr. Biol. **27**, R1287–R1292. (10.1016/j.cub.2017.10.044) 29207274

[63] Eshun-Wilson L *et al*. 2019 Effects of α-tubulin acetylation on microtubule structure and stability. Proc. Natl. Acad. Sci. **116**, 10366–10371. (10.1073/pnas.1900441116) 31072936 PMC6535015

[64] Piperno G, Fuller MT. 1985 Monoclonal antibodies specific for an acetylated form of alpha-tubulin recognize the antigen in cilia and flagella from a variety of organisms. J. Cell Biol. **101**, 2085–2094. (10.1083/jcb.101.6.2085) 2415535 PMC2114011

[65] Bhagwat S *et al*. 2014 Acetylated α-tubulin is reduced in individuals with poor sperm motility. Fertil. Steril. **101**, 95–104.(10.1016/j.fertnstert.2013.09.016) 24268707

[66] Fukuda T, Nagashima S, Abe T, Kiyonari H, Inatome R, Yanagi S. 2016 Rescue of CAMDI deletion‐induced delayed radial migration and psychiatric behaviors by HDAC6 inhibitor. EMBO Rep. **17**, 1785–1798. (10.15252/embr.201642416) 27737934 PMC5283595

[67] Yan J, Seibenhener ML, Calderilla-Barbosa L, Diaz-Meco MT, Moscat J, Jiang J, Wooten MW, Wooten MC. 2013 SQSTM1/p62 interacts with HDAC6 and regulates deacetylase activity. PLoS One **8**, e76016. (10.1371/journal.pone.0076016) 24086678 PMC3785417

[68] Yao YL, Yang WM. 2011 Beyond histone and deacetylase: an overview of cytoplasmic histone deacetylases and their nonhistone substrates. J. Biomed. Biotechnol. **2011**, 1–15. (10.1155/2011/146493) PMC3014693 21234400

[69] Liu N, Xiong Y, Li S, Ren Y, He Q, Gao S, Zhou J, Shui W. 2015 New HDAC6-mediated deacetylation sites of tubulin in the mouse brain identified by quantitative mass spectrometry. Sci. Rep. **5**, 16869. (10.1038/srep16869) 26581825 PMC4652237

[70] Saunders HAJ, Johnson-Schlitz DM, Jenkins BV, Volkert PJ, Yang SZ, Wildonger J. 2022 Acetylated α-tubulin K394 regulates microtubule stability to shape the growth of axon terminals. Curr. Biol. **32**, 614–630.(10.1016/j.cub.2021.12.012) 35081332 PMC8843987

[71] Li S *et al*. 2004 A map of the interactome network of the metazoan C. elegans. Science **303**, 540–543. (10.1126/science.1091403) 14704431 PMC1698949

[72] Malovannaya A *et al*. 2011 Analysis of the human endogenous coregulator complexome. Cell **145**, 787–799. (10.1016/j.cell.2011.05.006) 21620140 PMC3131083

[73] Liu H, Hu PW, Budhiraja S, Misra A, Couturier J, Lloyd RE, Lewis DE, Kimata JT, Rice AP. 2020 PACS1 is an HIV-1 cofactor that functions in Rev-mediated nuclear export of viral RNA. Virology **540**, 88–96. (10.1016/j.virol.2019.10.004) 31759187 PMC7335006

[74] Nair‐Gill E *et al*. 2021 Calcium flux control by Pacs1‐Wdr37 promotes lymphocyte quiescence and lymphoproliferative diseases. EMBO J. **40**, e104888. (10.15252/embj.2020104888) 33630350 PMC8090855

[75] Arnedo M *et al*. 2022 Molecular basis of the Schuurs–Hoeijmakers syndrome: what we know about the gene and the PACS-1 protein and novel therapeutic approaches. Int. J. Mol. Sci. **23**, 9649. (10.3390/ijms23179649) 36077045 PMC9456036

[76] Kanca O *et al*. 2019 De novo variants in WDR37 are associated with epilepsy, colobomas, dysmorphism, developmental delay, intellectual disability, and cerebellar hypoplasia. Am. J. Hum. Genet. **105**, 413–424. (10.1016/j.ajhg.2019.06.014) 31327508 PMC6699142

[77] Dietzl G *et al*. 2007 A genome-wide transgenic RNAi library for conditional gene inactivation in Drosophila. Nature **448**, 151–156. (10.1038/nature05954) 17625558

[78] Bellen HJ *et al*. 2011 The Drosophila gene disruption project: progress using transposons with distinctive site specificities. Genetics **188**, 731–743. (10.1534/genetics.111.126995) 21515576 PMC3176542

[79] Lee PT *et al*. 2018 A gene-specific T2A-GAL4 library for Drosophila. eLife **7**, e35574. (10.7554/elife.35574) 29565247 PMC5898912

[80] Perkins LA *et al*. 2015 The Transgenic RNAi Project at Harvard Medical School: resources and validation. Genetics **201**, 843–852. (10.1534/genetics.115.180208) 26320097 PMC4649654

[81] Ryder E *et al*. 2007 The DrosDel Deletion Collection: a Drosophila genomewide chromosomal deficiency resource. Genetics **177**, 615–629. (10.1534/genetics.107.076216) 17720900 PMC2013729

[82] Parks AL *et al*. 2004 Systematic generation of high-resolution deletion coverage of the Drosophila melanogaster genome. Nat. Genet. **36**, 288–292. (10.1038/ng1312) 14981519

[83] Cook RK, Christensen SJ, Deal JA, Coburn RA, Deal ME, Gresens JM, Kaufman TC, Cook KR. 2012 The generation of chromosomal deletions to provide extensive coverage and subdivision of the Drosophila melanogaster genome. Genome Biol. **13**, R21. (10.1186/gb-2012-13-3-r21) 22445104 PMC3439972

[84] Dunst S *et al*. 2015 Endogenously tagged Rab proteins: a resource to study membrane trafficking in Drosophila. Dev. Cell **33**, 351–365. (10.1016/j.devcel.2015.03.022) 25942626 PMC4431667

[85] Chen D, McKearin DM. 2003 A discrete transcriptional silencer in the bam gene determines asymmetric division of the Drosophila germline stem cell. Development **130**, 1159–1170. (10.1242/dev.00325) 12571107

[86] Wakimoto BT, Lindsley DL, Herrera C. 2004 Toward a comprehensive genetic analysis of male fertility in Drosophila melanogaster. Genetics **167**, s.167. (10.1534/genetics.167.1.207) PMC1470876 15166148

[87] Frappaolo A, Karimpour-Ghahnavieh A, Cesare G, Sechi S, Fraschini R, Vaccari T, Giansanti MG. 2022 GOLPH3 protein controls organ growth by interacting with TOR signaling proteins in Drosophila. Cell Death Dis. **13**, 1003. (10.1038/s41419-022-05438-9) 36435842 PMC9701223

[88] Hirst J, Sahlender DA, Choma M, Sinka R, Harbour ME, Parkinson M, Robinson MS. 2009 Spatial and functional relationship of GGAs and AP‐1 in Drosophila and HeLa cells. Traffic **10**, 1696–1710. (10.1111/j.1600-0854.2009.00983.x) 19847956

[89] Schindelin J *et al*. 2012 Fiji: an open-source platform for biological-image analysis. Nat. Methods **9**, 676–682. (10.1038/nmeth.2019) 22743772 PMC3855844

[90] Szafer-Glusman E, Fuller MT, Giansanti MG. 2011 Role of Survivin in cytokinesis revealed by a separation-of-function allele. Mol. Biol. Cell **22**, 3779–3790. (10.1091/mbc.e11-06-0569) 21865602 PMC3192858

[91] Varmark H, Llamazares S, Rebollo E, Lange B, Reina J, Schwarz H, Gonzalez C. 2007 Asterless is a centriolar protein required for centrosome function and embryo development in Drosophila. Curr. Biol. **17**, 1735–1745. (10.1016/j.cub.2007.09.031) 17935995

[92] Frappaolo A, Zaccagnini G, Riparbelli MG, Colotti G, Callaini G, Giansanti MG. 2025 Supplementary material from: PACS deficiency disrupts Golgi architecture and causes cytokinesis failures and seizure-like phenotype in Drosophila melanogaster. Figshare (10.6084/m9.figshare.c.7671293)39999877

